# Structural features and antioxidant activities of polysaccharides from different parts of *Codonopsis pilosula* var. *modesta* (Nannf.) L. T. Shen

**DOI:** 10.3389/fphar.2022.937581

**Published:** 2022-08-24

**Authors:** Li-Xia Li, Meng-Si Chen, Zi-Yu Zhang, Berit Smestad Paulsen, Frode Rise, Chao Huang, Bin Feng, Xing-Fu Chen, Ren-Yong Jia, Chun-Bang Ding, Shi-Ling Feng, Yang-Ping Li, Yu-Long Chen, Zhen Huang, Xing-Hong Zhao, Zhong-Qiong Yin, Yuan-Feng Zou

**Affiliations:** ^1^ Natural Medicine Research Center, College of Veterinary Medicine, Sichuan Agricultural University, Chengdu, China; ^2^ Key Laboratory of Animal Disease and Human Health of Sichuan Province, College of Veterinary Medicine, Sichuan Agricultural University, Chengdu, China; ^3^ School of Pharmacy, University of Oslo, Oslo, Norway; ^4^ Department of Chemistry, University of Oslo, Oslo, Norway; ^5^ Animal Nutrition Institute, Sichuan Agricultural University, Chengdu, China; ^6^ Key Laboratory of Crop Ecophysiology and Farming System in Southwest China, Ministry of Agriculture, College of Agronomy, Sichuan Agricultural University, Chengdu, China; ^7^ College of Life Science, Sichuan Agricultural University, Ya’an, China; ^8^ Institute of Ecological Agriculture, Sichuan Agricultural University, Chengdu, China; ^9^ Sichuan Academy of Forestry, Ecology Restoration and Conservation on Forestry and Wetland Key Laboratory of Sichuan Province, Chengdu, China

**Keywords:** *Codonopsis pilosula*, polysaccharides, structural elucidation, *Caenorhabditis elegans*, antioxidant activity

## Abstract

In this study, three acidic polysaccharides from different plant parts of *Codonopsis pilosula* var. *Modesta* (Nannf.) L. T. Shen were obtained by ion exchange chromatography and gel filtration chromatography, and the yields of these three polysaccharides were different. According to the preliminary experimental results, the antioxidant activities of the polysaccharides from rhizomes and fibrous roots (CLFP-1) were poor, and was thus not studied further. Due to this the structural features of polysaccharides from roots (CLRP-1) and aerial parts (CLSP-1) were the object for this study and were structurally characterized, and their antioxidant activities were evaluated. As revealed by the results, the molecular weight of CLRP-1and CLSP-1 were 15.9 kDa and 26.4 kDa, respectively. The monosaccharide composition of CLRP-1 was *Ara*, Rha, Fuc, Xyl, Man, Gal, GlcA, GalA in a ratio of 3.8: 8.4: 1.0: 0.8: 2.4: 7.4: 7.5: 2.0: 66.7, and *Ara*, Rha, Gal, GalA in a ratio of 5.8: 8.9: 8.0: 77.0 in for CLSP-1. The results of structural elucidation indicated that both CLRP-1 and CLSP-1 were pectic polysaccharides, mainly composed of 1, 4-linked galacturonic acid with long homogalacturonan regions. Arabinogalactan type I and arabinogalactan type II were presented as side chains. The antioxidant assay in IPEC-J2 cells showed that both CLRP-1 and CLSP-1 promoted cell viability and antioxidant activity, which significantly increase the level of total antioxidant capacity and the activity of superoxide dismutase, catalase, and decrease the content of malondialdehyde. Moreover, CLRP-1 and CLSP-1 also showed powerful antioxidant abilities in *Caenorhabditis elegans* and might regulate the nuclear localization of DAF-16 transcription factor, induced antioxidant enzymes activities, and further reduced reactive oxygen species and malondialdehyde contents to increase the antioxidant ability of *Caenorhabditis elegans*. Thus, these finding suggest that CLRP-1 and CLSP-1 could be used as potential antioxidants.

## 1 Introduction

*Radix Codonopsis* has been historically used both in medicines and food in China. *Codonopsis pilosula* (Franch.) Nannf, *C. pilosula* Nannf. var. *Modesta* (Nannf.) L. T. Shen and *C. tangshen* Oliv are officially recorded in the Chinese Pharmacopeia (Committee, 2020) as plant resources. They have gained increasing attention because of the various pharmacological activities, such as improving the respiratory system (Gao et al., 2018), antitumor (Chen and Huang, 2018), antihypertensive effects, and so on (Shin et al., 2020). However, rhizomes and fibrous roots of *C. pilosula* are discarded when processing because rhizomes are considered to have an emetic effect by the ancients and fibrous root are considered as redundant. However, modern studies have shown that the chemical compositions of root and rhizomes of *C. pilosula* are similar, the high performance liquid chromatography fingerprints have more than 96% similarity, and polysaccharide is one of the major constituents (Lei et al., 2015; Rui-yan et al., 2018). Aerial parts of *C. pilosula*, are the important part of *C. pilosula*, with quite a high yield but have not been used rationally, as they are abandoned as waste in the production areas. But they have multiple chemical components and physiological activities (Xiao et al., 2005; Xie et al., 2017; Chen et al., 2020), and two acidic polysaccharides from the stems of *C. pilosula* and *C. tangshen* had been demonstrated that could be resistance to oxidative stress *in vitro* in our previous study (Zou et al., 2020). Thus, it is important to explore the value of the aerial part from *C. pilosula* var. *Modesta* (Nannf.) L. T. Shen. Recent studies suggest that several pharmacological effects of *Radix Codonopsis* are associated with its polysaccharide fractions. *C. pilosula* polysaccharides had been proved that have antioxidant activity (Wu et al., 2020; Luan et al., 2021). However, they only exist in the study of free radical scavenging activity and cell antioxidant activity *in vitro*.

Oxidative stress has always been regarded as the cause of disease and also one of the main driving factors of aging and age-related disease (Rai et al., 2009; Freund et al., 2010; Chen and Zhong, 2014). Under Oxidative stress, the imbalance of reactive oxygen species (ROS) in living organisms could damage DNA, proteins, and lipid membranes on cells (Lushchak, 2014), relevant for various diseases including cancer (Leinonen et al., 2014; Townsend et al., 2014), diabetes (Drel and Sybirna, 2010; Yan, 2014), cardiovascular (Feoli et al., 2014; Mei et al., 2015), and neurodegenerative diseases (Ahmad et al., 2014; Kamat et al., 2014; Zhang and Tian, 2014). Many studies showed that plant polysaccharides could be considered as an antioxidant to increase antioxidant enzymes activities and further ameliorate oxidative damage, like *Ligusticum chuanxiong* polysaccharides (Huang et al., 2017), *Chlorella vulgaris* polysaccharides (Yu et al., 2019), *C. pilosula* and its stems polysaccharides (Zou et al., 2020; Zou et al., 2021b). However, polysaccharides from different parts of *C. pilosula* var. *Modesta* (Nannf.) L. T. Shen have potential antioxidant properties *in vitro* and *in vivo* and are still have never been characterized.

*Caenorhabditis elegans* is a free-living nematode that has been extensively utilized as an animal model for research involving in oxidative stress and aging. If well fed, newly hatched individuals pass through four larval stages (L1, L2, L3, L4) and reach the adult stage after 3 days (Frézal and Félix, 2015). Therefore, *C. elegans* is a short-life system, with a short growth cycle, and the genome has been completely sequenced. These characteristics make this organism an ideal model for oxidant stress and aging research. With the emergence of some mutant strains, the worm has been used in the studies of oxidative stress, such as *Panax notoginseng* polysaccharides (Feng et al., 2018), *Bergenia emeiensis* polysaccharides (Zeng and Feng, 2020), and showed the good effect. Therefore, this animal model was thought to be a good model for antioxidant studies in this project.

**FIGURE 1:**
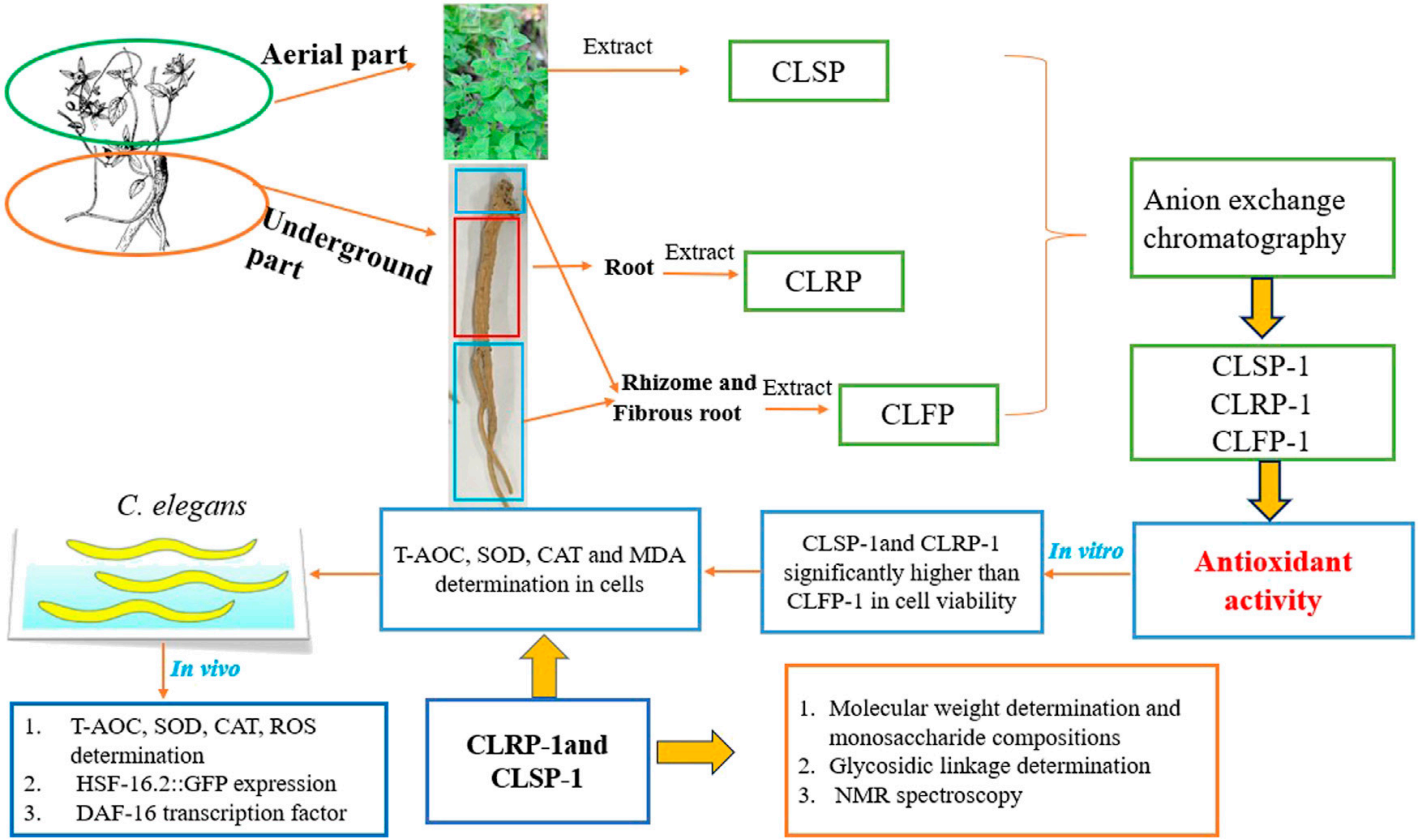
Schematic diagram of extraction, purification, structural characterization, and antioxidant activities of different parts of *C*. *pilosula* Nannf. var. *Modesta* (Nannf.) L. T. Shen.

This study aims to purify and characterize the acidic polysaccharides from the aerial part, root, rhizomes and fibrous root of *C. pilosula* Nannf. var*. modesta* (Nannf.) L. T. Shen, and compare the potential antioxidant activities (Figure 1). This is to reveal prospects for reasonable use of its rhizomes, fibrous root and aerial part, which are available in large quantities as agricultural waste in the *C. pilosula* Nannf. var*.modesta* (Nannf.) L. T. Shen production areas.

## 2 Materials and methods

### 2.1 Materials and reagents

*C*. *pilosula* Nannf. var. *modesta* (Nannf.) L. T. Shen was collected in Jiuzhaigou County (Tibetan Qiang Autonomous Prefecture of Ngawa, China) on 12 October 2019, and was identified by Yuan-Feng Zou, College of Veterinary Medicine, Sichuan Agricultural University. The plants were cultivated in high mountains with an altitude of around 2,500 m and had already been cultivated 3 years since seeding. The plants were divided into three parts, including root, rhizomes and fibrous root, and aerial parts, then were washed, dried, and pulverized to fine powder. All chemical reagents are analytical grade.

### 2.2 Extraction and purification of CLRP-1, CLFP-1 and CLSP-1

**FIGURE 2:**
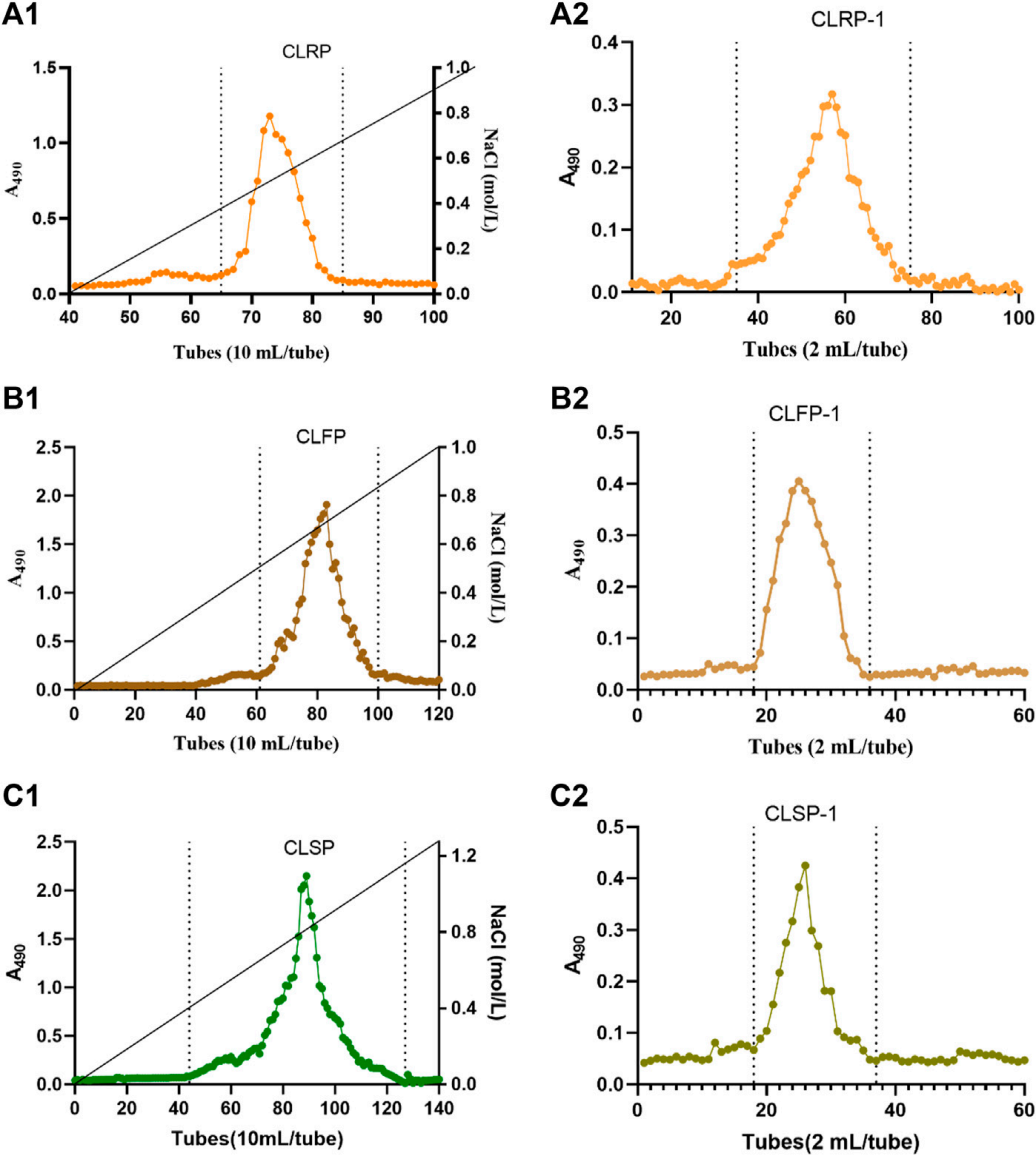
The elution profiles of CLRP, CLFP and CLSP. Elution curves of CLRP-1, CLFP-1 and CLSP-1 on DEAE anion exchange chromatography **(A1,B1,C1)** and purification of polysaccharide fractions of CLRP-1, CLFP-1 and CLSP-1 by gel filtration **(A2,B2,C2)**, respectively. A_490_ represents the absorbance at 490 nm by phenol-sulphuric acid method.

The plant material was extracted as described previously (Yu-Ping et al., 2018). Briefly, dried and powdered material (passed through 0.25 mm mesh) of each part (100 g) were extracted with refluxing 96% ethanol to remove low molecular weight and organic pigments, which was performed by 1-h extraction 6 times (v/w, 50 ml/g, 100°C, pH = 7.0), respectively. Then three crude polysaccharides were obtained by water extraction (v/w, 40 ml/g, 100°C, pH = 7) and alcohol precipitation, and they were extracted for two times, 2 hours every time. The crude polysaccharide from roots, rhizomes and fibrous roots, and aerial parts of *C. pilosula* Nannf. *var. modesta* L. T. Shen were named CLRP, CLFP, CLSP, respectively. The acidic polysaccharides were obtained by Diethylaminoethyl (DEAE)—Sepharose anion exchange column (50 mm × 40 cm, Beijing Rui Da Heng Hui Science Technology Development Co., Ltd. Beijing, China elution 25 C), were eluted with a linear NaCl gradient in water (0–1.5 M) at 2 ml/min, then dialysis and freeze-drying based on the previous studies (Zou et al., 2014; Zou et al., 2020), and named CLRP-1, CLFP-1, and CLSP-1 (Figures 2A1–2C1). The three acidic polysaccharide fractions were dissolved in 2 ml elution solution (10 mmol/L NaCl) and were filtered by 0.22 µm needle filter, respectively. Then the solutions were applied onto Hiload™ 26/60 Superdex™ 200 prep grade column (GE Healthcare) combined with the Äkta system for further purification (Figures 2A2–C2).

### 2.3 Molecular weight determination

The method referred to the literature adopted high performance size exclusion chromatography coupled with multi angle laser light scattering and refractive index detector (Wyatt Technology Co., Santa Barbara, CA, United States ) to detect the molecular weight of CLRP-1 and CLSP-1 (Wu et al., 2016). ShodexOHpak SB-806 M HQ (300 mm × 8.0 mm, id) column was used at 30°C to separated sample. The mobile phase was 0.9% NaCl aqueous solution at a flow rate of 0.5 ml/min. The injection volume was 100 μL. Data acquisition and analysis adopted Astra software (version 7.1.3, Wyatt Technology Co., Santa Barbara, CA, United States).

### 2.4 Determination of chemical composition and glycosidic linkage

The total polyphenols and total protein in CLRP-1 and CLSP-1 were quantitatively determined using the Folin-Ciocalteu (Chen et al., 2015) and Bradford protein assays (Bradford, 1976), respectively. The monosaccharide composition of CLRP-1 and CLSP-1 were determined by gas chromatography. The samples were treated with 3 M hydrochloric acid in MeOH for 20 h at 80°C. Then trimethylsilylated (TMS) derivatives of the methyl-glycosides were determined by gas chromatography. The method was the same as in the previous studies (Chambers and Clamp, 1971; Austarheim et al., 2012). Mannitol was used as internal standard. Finally, based on standards for all the monomers present, the monosaccharide compositions were identified and quantified.

Glycosidic linkage elucidation was performed by methylation study. First, the uronic acids in the polysaccharides were reduced with NaBD_4_ to their corresponding deuterated neutral sugars. Then methylation, hydrolysis, reduction and acetylation were carried out (Kim and Carpita, 1992; Zou et al., 2014). Briefly, 1 mg of dried sample was dissolved in DMSO, then the polysaccharide solution was mixed with DMSO/NaOH, and iodomethane was added to the polysaccharide solution to react. At the end of the reaction, the polysaccharide solution was mixed with ammonium hydroxide (2 M) and 50 μL NaBD_4_ (1 M) was mixed for 2.5 h, and finally the reaction was stopped with acetic acid then the sample was dried. The dried samples were washed by methanol twice to react with acetic anhydride, and finally the samples were mixed with dichloromethane, and the aqueous phase was discarded by centrifugation. The derivatives were analyzed by GC–MS using a GCMS-QP2010 (Shimadzu, Kyoto, Japan) attached to a Restek Rxi-5MS column. The injector temperature was 250°C, the detector temperature 300°C and the column temperature was 80°C when injected, then increased with 20°C/min to 170°C, followed by 0.5°C/min to 200°C and then 30°C/min to 300°C (Austarheim et al., 2012). Helium was used as the carrier gas. The retention time and integration in GC and the mass spectra of each peak were analyzed to characterize the methylated fragments.

### 2.5 Precipitation with the Yariv *β*-glucosyl reagent

Precipitation with the Yariv *β*-glucosyl (Yariv *β*-glucosyl, Future biotech, Beijing, China) reagent was performed on the samples as described by the previous method (Van Holst and Clarke, 1985). A colored precipitate would appear when Yariv *β*-glucosyl reagent was combined with a compound containing the AG-II structure. An aqueous solution of Arabic gum was used as a positive control.

### 2.6 NMR spectroscopy

10 mg of CLRP-1 and CLSP-1 were dissolved in 1 ml of D_2_O and freeze-dried, and then the above process was repeated to fully exchange active hydrogen. Afterward, samples were dissolved in D_2_O and were observed on Bruker AVIIIHD 800 instrument (Bruker, Fällanden, Switzerland) at a temperature of 60°C instrument to determine the ^1^H NMR spectrum with solvent suppression, ^13^C NMR, and HSQC, HMBC, and COSY spectra (Zou et al., 2021a).

### 2.7 Determination of antioxidant activity *in vitro*

#### 2.7.1 Cell and culture conditions

The intestinal porcine epithelial cells (IPEC-J2) were obtained from the Shanghai Institutes of Biological Sciences, Chinese Academy of Sciences (Shanghai, China). IPEC-J2 cells were routinely cultured in DMEM (Gibco Thermo Fisher Biochemical Inc. Beijing China). Supplemented with 10% FBS (Aus GeneX) and 1% penicillin–streptomycin (Invitrogen; Thermo Fisher Scientific, Inc. Waltham, MA, United States ) in an incubator under an atmosphere of 5% CO_2_ at 37°C.

#### 2.7.2 Cell viability assay

Cells were plated in 96-well cell plates (2 × 10^3^ cells/well); after 12 h the polysaccharide solutions and cell culture medium were mixed and supplied; the CCK-8 kit (CCK-8; Solarbio, Beijing, China) was used to detect cell viability. To induce oxidative stress, after the cells in a 96-well plate (2 × 10^3^ cells/well) were cultured for 24 h, the supernatant was carefully aspirated, 100 μL polysaccharide solutions (CLRP-1, CLFP-1, and CLSP-1) of different concentrations (20, 10, 5 μg/ml) were added and cultured for 12 h, and then 400 μM H_2_O_2_ was added and incubated for 12 h. Finally, aspirating the supernatant, CCK-8 and DMEM at a ratio of 1:10 were mixed and added to a 96-well plate, which was incubated at 37°C for 1 h, and then the absorbance was detected at 450 nm (Huang et al., 2017).

#### 2.7.3 Determination of total antioxidant capacity, malondialdehyde and antioxidant enzyme activities in IPEC-J2

Cells were plated in 6-well cell plates. According to the previous results of the cell viability assay in the experiment, the activity of CLFP-1 was very poor, so only CLRP-1 and CLSP-1 were used for subsequent experiments. Polysaccharide solutions (20, 10, 5 μg/ml) were supplemented and co-cultured for 12 h, H_2_O_2_ was added into the cells, and then the cells were collected for detection of enzyme activity. The total protein content was detected using the total protein assay kit (BCA; Solarbio, Beijing, China). The levels of total antioxidant capacity (T-AOC), superoxide dismutase (SOD), catalase (CAT), and malondialdehyde (MDA) were measured with ELISA assay kits (ELISA assay kits; mlbio, Shanghai, China).

### 2.8 Determination of antioxidant activity *in vivo*

#### 2.8.1 The source of *C. elegans* strains and culture condition

Wild-type N2, *C. elegans*, TJ375 (gpIs1 [*hsp-16.2*p::GFP]), *C. elegans*, TJ356 DAF-16::gfp (*zIs356* (pDAF-16::DAF-16-GFP; *rol-6*), *C. elegans* and *Escherichia coli* OP50 strains were obtained from the *Caenorhabditis* Genetics Center (CGC). The nematodes were cultured on nematode growth medium (NGM) plates at 20°C with OP50 bacteria as a food source (Feng et al., 2018).

#### 2.8.2 Reproductive toxicity determination

Synchronized offspring of worms were obtained by laying eggs in 2 h until growing L4 stage. Then randomly selected 10 worms were transferred to fresh NGM medium. Each worm had a separate medium. Different doses of polysaccharide solution (0.75, 0.25 mg/ml) were added to OP50 to make the worms lay eggs on the medium. Transfer to a fresh NGM medium every 24 h until no eggs were produced. Finally, the number of eggs within 24 h of *C. elegans* was counted.

#### 2.8.3 Reactive oxygen species level determination

Synchronized L1 larvae were cultured at 20°C to the L4 stage. Afterward, thermal stress was performed, and the worms were washed into the Eppendorf, and then tested with the ROS kit (Beyotime Biotechnology, Shanghai, China) according to its instructions. In brief, added S-Basal buffer and 2',7'-dichlorodihydrofluorescein diacetate to the Eppendorf at a ratio of 1:1,000. After being incubated for 30 min in the dark, which washed 3 times and left a small amount of liquid in the Eppendorf. Finally, the worms were anesthetized, observed, and photographed under a fluorescence microscope (OLYMPUS, Tokyo, Japan). There were at least 10 worms in each concentration.

#### 2.8.4 Assay of T-AOC, malondialdehyde and antioxidant enzyme activities in *C. elegans*

Synchronized L1 larvae were inoculated into NGM medium, which contained OP50 and polysaccharides grown to L4 stage. Then the worms were placed in a constant temperature incubator at 35°C for 4 h. Afterward the worms were incubated at 20°C for 2 h. Finally, the worms were collected to detect the content of total protein, T-AOC, SOD, and CAT by kit (Nanjing Jiancheng Biotechnology Institute, China). The detection steps were operated according to the kit instructions.

### 2.9 Gene expression level determination

#### 2.9.1 Visualization of the expression of HSP-16.2::GFP

TJ375 strain contains a reporter transgene that expresses a green fluorescence protein (GFP) regulated by a promoter of small heat shock proteins (Guerrero-Rubio et al., 2020). The synchronized worms grew on the plate in the absence and presence of CLSP-1 to L4 stage. After thermal stress (35°C, 1 h), the expression of HSP-16.2:: GFP was quantified by observing the fluorescence intensity of the worms by using a fluorescence microscope, and there were at least 15 worms in each concentration.

#### 2.9.2 Visualization of the expression of DAF-16:: GFP

TJ356 strain carried a GFP on the transcription factor of DAF-16, making the visualization of DAF-16 clear. After thermal stress (35°C, 1 h), the location of the transcription factor of DAF-16 was observed by using a fluorescence microscope and there were at least 10 worms in each concentration.

### 2.10 Statistical analysis

All relative fluorescence intensities were processed by the software Image-J (National Institutes of Health, Bethesda, MD, United States), and all values were expressed as mean ± standard deviation (SD). All experiments were repeated 3 times, and the data were analyzed by IBM SPSS statistic software (Version 25.0, United States).

## 3 Results and discussion

### 3.1 Extraction and fractionation of polysaccharide

The crude polysaccharides from the three parts of *C. pilosula* Nannf. var*.modesta* (Nannf.) L. T. Shen were obtained from water extracts after ethanol precipitation. The yield of CLRP, CLFP and CLSP were 20.5% (20.5 g CLRP from 100.0 g dried roots), 16.3% (16.3 g CLFP from 100.0 g dried rhizomes and fibrous roots) and 7.9% (7.9 g CLSP from 100.0 g dried aerial parts), respectively. The acidic polysaccharide fractions were obtained after ion exchange separation and gel filtration, named CLRP-1, CLFP-1, and CLSP-1, as shown in Figures 2A1–C1. Their yields were 15.0% (0.24 g CLRP-1 from 1.60 g CLRP), 18.1% (0.29 g CLFP-1 from 1.60 g CLFP) and 30.0% (0.48 g CLSP-1 from 1.60 g CLSP), respectively. Under the same extraction and purification process, it was observed that the yield of CLRP was slightly higher than CLFP. And CLSP was the lowest among the three crude extracts, whereas the yield of CLSP in three acidic polysaccharide fractions was the highest. Interestingly, CLRP was the highest yield among the three crude extracts, but CLRP-1 was the lowest yield of the three acidic polysaccharide fractions. Compared with our previous study, the yield of CLSP (7.9%) was higher than from the stems of *C. pilosula* (CPSP, 4.6%), while lower than that from stems of *C. tangshen* (CTSP, 8.5%) (Zou et al., 2020). For roots, the yield of CLRP (20.5%) is lower than CTP (22.8%), and almost the same as CPP (20.3%) (Zou et al., 2021b). Regarding the purified acidic fraction, the yield of CLSP-1 was lower than that from stems of *C. pilosula* (CPSP-1) and *C. tangshen* (CTSP-1) (Zou et al., 2020). And CLRP-1 (14.73%) was lower than that from the root of *C. pilosula* (CPP-1, 31.3%) and *C. tangshen* (CTP-1, 30.3%) (Zou et al., 2021b). It indicated that the yield of polysaccharides from *Codonopsis* plant varies based on the species and plant parts.

### 3.2 Molecular weight determination and chemical compositions

High-efficiency Size Exclusion Chromatography combined with Multi-Angle Laser Light Scattering (SEC-MALLS) and the refractive index was used to detect molecular weight. Both CLRP-1 and CLSP-1 were homogeneous fractions as shown in Figures 2B1, B2. The molecular weight of CLRP-1 and CLSP-1 is estimated to be 15.9 kDa and 26.4 kDa, respectively. The *Mw* of CLSP-1 was higher than polysaccharides from the stems of *C. pilosula* (CPSP-1, 13.1 kDa) and *C. tangshen* (CTSP-1, 23 kDa) (Zou et al., 2020). Polysaccharides from *C. pilosula* Nannf. var. *modesta* (Nannf.) L. T. Shen (CPP1a,1b,1c), had a higher molecular weight than CLSP-1 (Yang et al., 2013; Zhang et al., 2017; Bai et al., 2018). The *Mw* of CLSP-1 was lower than most of the polysaccharides from the root of *C. pilosula*. Compared with polysaccharides from *C. pilosula* Nannf. var. *modesta* L. T. Shen roots (100 WCP-II-I), CLSP-1 was much lower than 100 WCP-Ⅱ-Ⅰ (53.2 kDa) (Zou et al., 2014). Compared with CTP-1, CPP-1 and 100 WCP-II-I, CLRP-1 had the lowest *Mw* (Zou et al., 2014; Zou et al., 2021b). These differences may be due to the different material sources and plant species, as well as different extraction methods. Additionally, the *Mw* of CLSP-1 was higher than CLRP-1, which also demonstrated that the different part of a plant was another critical factor affecting the *Mw* of polysaccharides.

As shown in Table 1, similar amount of polyphenol and protein were found in both CLRP-1 and CLSP-1, which indicated those two fractions were phenolic and protein contained polysaccharides (Capek and Košťálová, 2022). The monosaccharide compositions of the CLRP-1 and CLSP-1 were investigated by GC as the TMS ramifications of the methyl-glycosides. As shown in Table 1, there are some diversities in monosaccharide composition between CLRP-1 and CLSP-1. The CLSP-1 consists of arabinose (Ara), rhamnose (Rha), galactose (Gal) and galacturonic acid (GalA), with the ratio of 5.8: 8.9: 8.0: 77.0 (mol%). However, compared with CLSP-1, CLRP-1 had more abundant components, and the compositions of fructose (Fuc), xylose (Xyl), mannose (Man), glucose (Glc), glucose acid (GlcA) only exist in CLRP-1. This result showed that GalA was the main monosaccharide composition of CLRP-1 and CLSP-1, which were both typical pectic polysaccharides (Table 1). The contents of Ara, Rha and Gal in CLSP-1 were lower than those in CTSP-1 and CPSP-1; CLSP-1 contains higher amounts of GalA. Compared with 100WCP-II-I, CLRP-1 had similar monosaccharide composition, but CLSP-1 had no xylose (Xyl), glucose (Glc) and Glucuronic acid (GlcA). 100 WCP-II-I had higher amounts of Ara and Gal and a lower amount of Rha than CLSP-1 and CLRP-1 (Zou et al., 2014). The structure of CLSP-1 and CLRP-1 was different from other *C. pilosula* polysaccharides reported in previous studies, such as CPP1a, CPP1b, CPP1c. Monosaccharide composition analysis showed that CPP1a contained Rha, Ara, Glc, Gal and GalA in a molar ratio of 1.3:12.3:3.5:10.4:1.2 (Bai et al., 2018). The result indicated that compared with CLSP-1 and CLRP-1, there were various amounts of monosaccharides and proportions. Compared with CPP1b and CPP1c, CLRP-1 and CLSP-1 both had a higher amount of GalA (Yang et al., 2013; Zhang et al., 2017). It was proved that the polysaccharides from different parts of *C. pilosula* Nannf. var*.modesta* (Nannf.) L. T. Shen were all pectic polysaccharides, but their structures were diverse.

**TABLE 1:** Monosaccharide compositions (mol%) and *Mw* of polysaccharide fraction from the different plant parts of *C. pilosula* Nannf.var.*modesta* (Nannf.) L.T.Shen (CLSP-1).

Monosaccharides	CLRP-1	CLSP-1
Ara	3.8	5.8
Rha	8.4	8.9
Fuc	1.0	n.d
Xyl	0.8	n.d
Man	2.4	n.d
Gal	7.4	8.0
Glc	7.5	n.d
GlcA	2.0	n.d
GalA	66.7	77.0
Total carbohydrate (%)	81.1	80.0
Total polyphenol (%)	2.5	3.5
Total protein (%)	3.8	4.3
Mw (kDa)	15.9	26.4

### 3.3 Glycosidic linkage determination

The glycosidic linkage was identified using GC-MS after methylation of CLRP-1and CLSP-1 fragments. As shown in Table 2, the 1,4-linked galacturonic acid units were 66.7% and 71.7% of the monosaccharide content, which indicated that a homogalacturonan (HG) backbone could be present and that the two polymers were both typical pectin polysaccharide (Shakhmatov et al., 2020), and CLSP-1 had longer HG than CLRP-1. 1,4-linked GalA with 1,2 and 1,2,4-linked Rha indicated that RG-I regions were located on position four of the Rha units as side chains in CLRP-1 and CLSP-1 (Paulsen and Barsett, 2005). These chains consisting mainly of 1,4-Gal with arabinose terminally were typical for the chains normally termed arabinogalactan I (AG-I) (Yu et al., 2010; Ho et al., 2016). In addition, a trace amount of 1,3-linked, 1,6-linked and 1,3,6-linked Gal indicated the presence of arabinogalactan type II (AG-II) side chains presented in CLRP-1 and CLSP-1, like previous studies of PLBP-II, PLBP-II-I, CTSP-1, CPP-1, and CTP-1 (Yao et al., 2018; Zou et al., 2020; Zou et al., 2021b). The positive precipitation with the Yariv β-glucosyl reagent test also verified the presence of AG-II in those two polysaccharide fractions (Paulsen et al., 2014). The AG-II constituted a minor part of the chains attached to the RG-I region. Moreover, compared with CPSP-1 and CTSP-1, CLSP-1 had a higher amount of 1,4-linked GalA*p* than CPSP-1 and CTSP-1, which indicated that CLSP-1 had the longest homogalacturonan (HG) backbone among the three pectic polysaccharides (Zou et al., 2020). According to the number of arabinose units, CLSP-1 had lower amounts of branches than CPSP-1 and CTSP-1, but it was higher than CLRP-1 (Zou et al., 2020). CLSP-1 contained typical AG-Ⅱ linkages, and the content of AG-Ⅱ linkages were higher than CLRP-1, but lower than CPSP-1 and CTSP-1 (Zou et al., 2020). In CLSP-1, the amount of AG-I side chains was higher than that fraction CLRP-1 and CTSP-1, and it was not detected in CPSP-1. Compared with 100 WCP-II-I, CLSP-1 had a higher amount of typical AG-II linkages and a lower amount of typical AG-I linkages, which suggested that AG-II structures were the main part of the side chains attached to the RG-I region in CLSP-1. AG-I structures were the main part of the side chains in 100 WCP-II-I (Zou et al., 2014). The types of glycosidic linkage existing in CLRP-1 were different from the reported studies about roots, CLRP-1 had more types of glycosidic linkage than CTP-1 and CPP-1, indicating that CLRP-1 had more branching structures, such as 1,5-linked Ara*f*, 1,3,5-linked Ara*f*, 1,3-linked Rha*p*, 1,6*-*linked Gal*p*, 1,3,4-linked Gal*p* and 1,3,4-linked Gal*p*A (Zou et al., 2021b). The results demonstrated that the polysaccharides from different plant parts of *C. pilosula* Nannf. var*.modesta* (Nannf.) L. T. Shen and different varieties of *C. pilosula* roots had different glycosidic linkage units compared with the published reports.

**TABLE 2:** The linkage types (mol%) of the monosaccharides present in the fractions CLRP-1 and CLSP-1 by GC-MS after methylation.

	Linkage type	CLRP-1	CLSP-1
Ara	Tƒ	2.4	3.5
	1→2ƒ	n.d	1.1
	1→3ƒ	n.d	0.8
	1→5ƒ	1.0	n.d
	1→3, 5ƒ	0.9	0.5
Rha	T*p*	0.7	4.5
	1→2*p*	3.6	0.6
	1→2, 4*p*	2.5	3.8
	1→3*p*	3.0	n.d
Gal	T*p*	1.8	0.5
	1→4*p*	1.1	2.9
	1→3*p*	1.5	1.4
	1→6*p*	0.8	1.0
	1→3, 6*p*	1.4	2.2
	1→3, 4*p*	0.5	n.d
Xyl	T*p*	0.6	n.d
Glc	T*p*	1.4	n.d
	1→6*p*	1.4	n.d
	1→4*p*	4.8	n.d
Man	1→2*p*	2.2	n.d
Fuc	T*p*	1.2	n.d
GalA	T*p*	3.5	1.4
	1→4*p*	59.8	71.7
	1→3, 4*p*	1.9	3.4
GlcA	1→4*p*	1.1	n.d

### 3.4 NMR spectroscopy analysis

**FIGURE 3:**
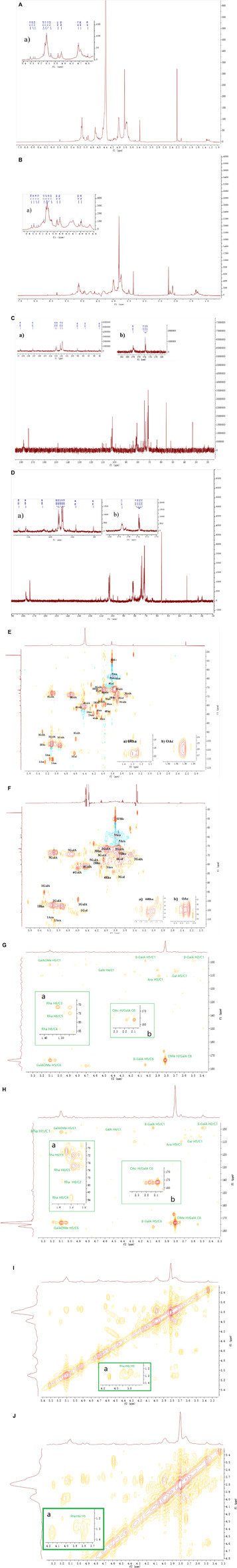
The ^1^H and ^13^C NMR spectra and HSQC NMR spectra of CLRP-1 and CLSP-1. **(A,B)** ^1^H NMR spectra of CLRP-1 and CLSP-1, respectively, the inset plots were the zoomed of **(a)** 1H region of ^1^H NMR (δ 4.5–5.5 ppm). **(C,D)** ^13^C NMR spectra of CLRP-1 and CLSP-1, respectively, the inset plots were the zoomed of **(a)** 1C region of ^13^C-NMR spectra (δ 95–115 ppm) and **(b)** downfield region of ^13^C-NMR spectra (δ 170–180 ppm). **(E,F)** HSQC NMR spectra of CLRP-1 and CLSP-1, the inset plots were the zoomed of **(a)** methyl group of rhamnose. **(b)** *O*-acetyl groups (*O*Ac). **(G,H)** HMBC NMR spectra of CLRP-1 and CLSP-1; **(a)** correlation of H6 to carbon of Rha in HMBC spectra; **(b)** correlation of proton of *O*Ac to C6 of GalA in HMBC spectra. **(I,J)** COSY NMR spectra of CLRP-1 and CLSP-1, **(a)** correlation between proton of Rha in COSY spectra.

1D and 2D NMR were applied to further confirm the sugar residues of CLRP-1 and CLSP-1. The signals of sugar residues were assigned according to the chemical shifts and combined with the literature values (Habibi et al., 2004; Kang et al., 2011; Košťálová et al., 2013; Chen et al., 2017; Du et al., 2018; Jiao et al., 2018; Shakhmatov et al., 2018; Yao et al., 2018; Huang et al., 2019; Liu et al., 2020; Shakhmatov et al., 2020). Some signals with trace amounts were not detected. The ^1^H and ^13^C NMR spectra of CLRP-1 and CLSP-1 were shown in Figure 3; Table 3. The EE, GG, EG, and GE represent diads of two methyl-esterified Gal*p*A residues; diads of two non-esterified Gal*p*A residues; diads of both methyl-esterified and non-esterified galacturonic acid residues, respectively (Košťálová et al., 2013).

**TABLE 3:** ^1^H and ^13^C chemical shifts (ppm) of polysaccharide fraction of CLRP-1 and CLSP-1.

Linkage type	H1/C1	H2/C2	H3/C3	H4/C4	H5/C5	H6/C6	OMe	OAc
CLRP-1								
→4-α-D-Gal*p*A	5.30/94.9	3.75/70.9	3.99/71.3	4.47/80.8	4.41/73.3	/177.6		
-4-β-D-Gal*p*A-(1-	4.59/99.0	3.50/74.5	3.76/75.8	4.43/80.5	4.13/79.0	/178.0		
EG: -4-α-D-Gal*p*A-(1-	5.10/101.7	3.75/70.9	3.99/71.3	4.43/80.5	4.70/74.0	/178.0		2.09/22.7
GE: -4-α-D-Gal*p*A-(1-	5.12/101.7	3.75/70.9	3.99/71.3	4.43/80.5	4.70/74.0	n.d		
EE: -4-α-D-GalA*p*6Me-(1-	4.92/102.6	3.88/69.3	3.99/71.3	4.43/80.5	5.10/73.1	/173.6	3.81/55.1	
EG: -4-α-D-Gal*p*A6Me-(1-	4.95/102.6	3.88/69.3	3.99/71.3	4.43/80.5	5.05/73.1	/173.7	3.81/57.1	
-2,4)-α-Rha*p*-(1-	5.27/101.0	#FF0000	#FF0000	#FF0000	#FF0000	1.24/19.1		
		4.13/79.0	4.14/71.1	3.86/83.9	3.74/75.3	1.30/19.4		
T-α-L-Ara*f*-(1	5.08/110.2	4.13/83.7	3.95/79.4	4.04/86.6	3.79/63.4			
-5-α-L-Ara*f*-(1-	5.25/111.8	4.13/83.7	4.00/79.6	4.14/86.8	3.81/63.7			
-3,5-α-L-Ara*f*-(1-	5.15/109.8	4.13/83.7	3.93/76.3	4.09/85.3	3.81/63.7			
-3)-β-Gal*p*-(1-	4.45,4.46/105.5	3.68/74.7	3.86/84.2	4.26/68.4	n.d	3.79/63.4		
-4-β-D-Gal*p*-(1-	4.62/107.1	3.68/74.7	3.76/75.8	4.13/79.0	3.70/77.4	3.80/69.1		
CLSP-1								
→4-α-D-Gal*p*A	5.30/95.0	3.74/71.0	3.98/71.4	4.45/81.1	4.43/73.2	/177.2		
-4-β-D-Gal*p*A-(1-	4.59/99.1	3.48/74.6	3.65/75.2	4.45/81.1	4.05/77.3	/177.5		
α-Gal*p*A-(1→	5.03/102.8	3.72/n.d	3.89/72.2	4.40/81.4	4.71/74.2	/177.7		
EE: -4-α-D-GalA*p*6Me-(1-	4.91/103.0	n.d	3.98//71.4	4.36/80.6	5.10/73.4	/173.7	3.81/55.2	
EG: -4-α-D-Gal*p*A6Me-(1-	4.95/103.0	n.d	3.98//71.4	4.36/80.6	5.17/73.9	/173.5	3.81/55.2	
GE: -4-α-D-Gal*p*A-(1-	5.12/102.2	3.74/71.0	3.98/71.4	4.48/81.1	4.71/74.3	n.d		
GG:-4-α-D-Gal*p*A-(1	5.15/102.3	3.74/71.0	3.98/71.4	4.45/81.1	4.71/74.3	/177.5		2.14/23.0
								2.09/23.0
-2, 4-α-L-Rha*p*-(1-	5.27/100.3	#FF0000	#FF0000	#FF0000	#FF0000	1.24/19.4		
		4.13/78.8	4.14/71.2	3.82/83.8	3.74/75.2	1.29/19.6		
T-α-L Rha*p*-(1-	5.34/102.5	4.05/77.3	n.d	3.74/71.0	n.d	1.37/20.0		
T-α-L-Ara*f*-(1→	5.26/112.3	4.12/84.0	4.05/77.3	4.05/n.d	3.74/63.4			
→3,5)-α-Ara*f*-(1→	5.10/110.6	n.d	4.13/84.2	4.13/n.d	3.61/69.2			
-4-β-D-Gal*p*-(1-	4.62/107.0	n.d	3.65/75.2	n.d	3.69/78.0	3.81/64.0		

In the ^1^H NMR spectra, signals ranging 10 to 6 ppm is known to contain proton resonances of the amide group in the polypeptide chain (Shakhmatov et al., 2016), in the present study, singal around 6.11 ppm was found in both fractions, which further proved the presence of protein in both fractions. Signal at 5.80 ppm in both fractions was assigned to polyphenols, which may connected onto polysaccharide fractions (Capek and Košťálová, 2022). In the ^13^C NMR spectra (Figures 3C, D), signal at 33.1 ppm were found in both fraction, which may assign to amino acid of the protein in the samples, such as Phenylalanine and Valine. And signals around 143–147 ppm in both fractions, were assigned to carbon signals of polyphenols, such as ferulic acid. Those results indicated that both polysaccharides were polyphenols and protein contained polysacchrides, this was similar to other cell wall polysaccharides reported by other studies (Capek and Košťálová, 2022; Shakhmatov et al., 2016).

The ^1^H NMR spectrum of fraction CLRP-1 contains twelve main peaks in the anomeric region, which were signed in Figure 3A. The intense signals of H/C-atoms at 5.30/94.9, 4.59/99.0, 5.10/101.7, 5.12/101.7 ppm belong to →4-α-D-Gal*p*A, -4-β-D-Gal*p*A-(1-, while that at 4.84/103.0 ppm belongs to the methyl esterified EE: -4-α-D-Gal*p*A6Me-(1- and EG: -4-α-D-Gal*p*A6Me-(1- (Košťálová et al., 2013; Zou et al., 2021b). In the downfield region of ^13^C NMR spectrum, the signal at 178.0 ppm was assigned to GE: -4-α-D-Gal*p*A-(1- and -4-β-D-Gal*p*A-(1-, that at 173.7 and 173.6 ppm were assigned to the carboxyl groups of methyl esterified EE: -4-α-D-Gal*p*A6Me-(1- and EG: -4-α-D-Gal*p*A6Me-(1-. The intense signals of H/C-atoms at 3.81/55.1, 57.1 ppm in the HSQC spectrum suggest that CLRP-1 had the methyl esterified Gal*p*A units (*OMe* in Figure 3E) (Zou et al., 2020). The cross peak at 5.27/101.0 ppm was assigned to -2,4-α-L-Rha*p*-(1→ and this at 1.30/19.4 ppm belongs to the methyl group of the Rha*p* glycosidic linkages (6Rha*p* in Figure 3Ea) (Chen et al., 2017; Zou et al., 2021b). The cross peaks in HMBC (Figure 3G) and COSY spectra (Figure 3I), further prove the assignment of Rha*p* in Table 3. The cross peaks at 5.15/109.8, 5.08, 5.10/110.2 ppm and 5.25/111.8 ppm were assigned to T-α-L-Ara*f*-(1-, -5-α-L-Ara*f*-(1- and →3,5)-α-L-Ara*f*-(1→ (Zou et al., 2021b). The signals of H/C-atoms at 4.45, 4.46/105.5 ppm and 4.62/107.1 ppm in the HSQC spectrum confirmed -3-β-D-Gal*p*-(1-and -4-β-D-Gal*p*-(1-, as same as shown in Table 3 (Zou et al., 2020; Zou et al., 2021b). In addition, the cross peak 2.09/22.7 ppm indicated that CLRP-1 had the acetylated 2-*O*- and/or 3-*O-* 1,4-α-D-Gal*p*A (*O*Ac in Figure 3Eb).

Typically, the ^1^H NMR spectrum indicated that CLSP-1 mainly contained 12 signals in anomeric protons region (4.6–5.5 ppm, Figure 3Ba). The signals at *δ* 5.30 and 5.03 ppm, were assigned to →4-α-D-Gal*p*A, α-Gal*p*A-(1→, respectively. The strong anomeric proton signals at *δ* 5.10 ppm were assigned to →3,5)-α-Ara*f*-(1→, and the signal at *δ* 5.14 and *δ* 5.26 ppm corresponded to T-α-Ara*f*-(1→. The signals at *δ* 5.34, 5.27 ppm corresponded to T-α-L Rha*p*-(1-, -2, 4-α-L-Rha*p*-(1-. The assignment of -2, 4-α-L-Rha*p*-(1- were according to the HMBC (Figure 3H) and COSY spectra (Figure 3J). The signals at *δ* 4.59, 4.62 ppm were assigned to T-α-L-Gal*p*-(1- and -4-β-D-Gal*p*-(1-(Zou et al., 2020). The chemical shift signals at *δ* 1.20 ppm illustrated the occurrence of the methyl group in Rha*p* residues (Tada et al., 2009). In ^13^C NMR spectrum (Figure 3D), the signal at *δ* 177.3 ppm was assigned to the carboxyl group of →4-α-D-Gal*p*A without methyl esterified, and the signal at *δ* 177.5 ppm was assigned to the carboxyl group of -4-α-D-Gal*p*A-(1, -4-β-D-Gal*p*A-(1- without being methyl esterified. The signal at *δ* 173.5 ppm was assigned to the carboxyl group of -4-α-D-Gal*p*A6Me-(1-with methyl esterification. The methyl and acetyl ester contents were calculated from the integration of ^1^H NMR. Integration of signals at *δ* 4.70 ppm that indicated H5 of non-esterified α-D-Gal*p*A). Integration of signals at *δ* 4.91 ppm and *δ* 4.95 ppm, they were H1 of esterified α-D-Gal*p*A, and *δ* 5.10 ppm contains H1 of non-esterified α-D-Gal*p* A and H5 of esterified α-D-Gal*p*A. Integration of signals at *δ* 3.80 ppm indicated *O*Me of α-D-Gal*p*A. Integration of signals at *δ* 2.10 ppm demonstrated the *O*Ac of α-D-Gal*p*A. The above integral value indicated a ratio between methyl esterified α-D-Gal*p*A and acetyl ester esterified α-D-Gal*p*A of about 1:10 and 1:4 in CLRP-1 and CLSP-1, respectively. Thus, there is a higher DA in CLSP-1 (5.88%) than CLRP-1 (2.33%), and the DM of the two polysaccharides was 23.27% (CLRP-1) and 23.52% (CLSP-1). The H6/C6 of Rha*p* was found around *δ* 1.21/17.42 ppm (Figure 3Fa). The cross peak at *δ* 2.14/23.04 ppm in the HSQC spectrum represented *O*-acetyl groups (*O*Ac Figure 3Fb), and the cross peak at *δ* 3.81/55.21 ppm indicated that it was methyl esterified. According to the HSQC spectra, -4-α-D-Gal*p*A-4-α-D-Gal*p*A-(1, -4-α-D-Gal*p*A6Me-(1, -4-β-D-Gal*p*-(1- was detected, and it had the structure of AG-I and AG-II, which was a typical pectin polysaccharide structure. It was consistent with the results of the stems of the *C. pilosula* and *C. tangshen* and *C. pilosula* polysaccharides from the root in our previous studies (Zou et al., 2014; Zou et al., 2020). According to Table 3; Figure 3, all data above demonstrated that an important feature of the fractions CLRP-1 and CLSP-1 are RG-I backbone, with branching AG-I and AG-II side chains on position four of Rha*p*. This was consistent with a highly ramified pectic polysaccharide CTSP-1 and 100 WCP-II-I (Zou et al., 2014; Zou et al., 2020). This proposed structure was based on a normal characteristic of pectin, but the exactly branching position cannot be determined.

### 3.5 CLRP-1 and CLSP-1 protect IPEC-J2 cells against oxidative stress

**FIGURE 4:**
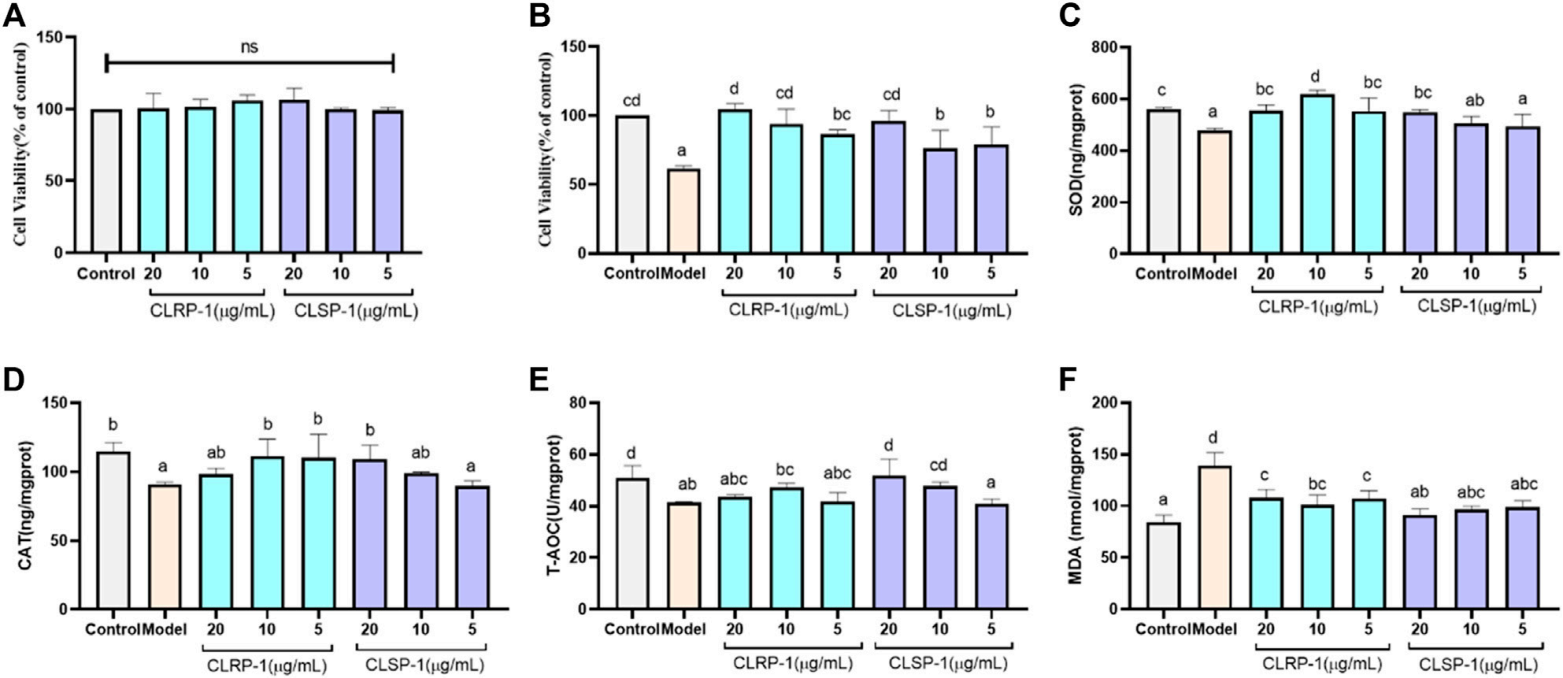
CLRP-1 and CLSP-1 promote antioxidant defense of IPEC-J2 cells. **(A)** CLRP-1 and CLSP-1 have no proliferative toxicity to normal IPEC-J2 cells. **(B)** The effects of different concentrations of CLRP-1 and CLSP-1 treated cells for 12 h, then by 400 μM H_2_O_2_ co-culture for 12 h on the proliferation and viability of IPEC-J2 cells. **(C)** Quantification illustrates the level of SOD. **(D)** Quantification illustrates the level of CAT. **(E)** Quantification illustrates the level of T-AOC. **(F)** Quantification illustrates the level of MDA. Bars with different lowercase letters **(a–d)** are significantly different (*p* < 0.05).

The antioxidant activity of two polysaccharide fractions, CLRP-1 and CLSP-1, were evaluated in the IPEC-J2 cell line. It can be used for intestinal antioxidant defense, which has the advantage of being directly comparable to the experimental animal that is used as an in vitro model for humans. Among all non-primates, the gastrointestinal tract of pigs is the most suitable *in vitro* model and is also used for oxidative stress and drug screening studies (Deglaire and Moughan, 2012; Saaby et al., 2016; Guo et al., 2019; Zhuang et al., 2019). As shown in Figure 4A, there was no significant effect on cell viability of IPEC-J2 cells after giving different doses of CLSP-1 and CLRP-1, indicating that both these two fractions have no cytotoxicity. The cell viability of IPEC-J2 cells was decreased significantly (*p* < 0.01) after H_2_O_2_-treatment, suggesting that the model was successful. After co-cultured with CLRP-1 and CLSP-1 for 24 h, the cell viability was significantly higher than the model group, as shown in Figure 4B. Therefore, it was proposed that CLRP-1 and CLSP-1 both have a certain protective effect against oxidative stress *in vitro*.

To give more evidence that CLRP-1 and CLSP-1 could prevent the H_2_O_2_-induced oxidative stress, the levels of the indicators including SOD, CAT, T-AOC, MDA were analyzed in IPEC-J2 cells. As shown in Figure 4, the activities of all antioxidant enzymes (SOD, CAT) and T-AOC in the H_2_O_2_ treatment group were lower than the control group; while the level of MDA was significantly increased (Figures 4C–F). The activities of SOD and CAT were significantly higher than that in the H_2_O_2_-treated group after giving CLRP-1 and CLSP-1, whereas CLRP-1 had a better effect than CLSP-1 (Figures 4C,D). And in the polysaccharides-pretreated group, T-AOC demonstrated an increasing trend even if there was no dramatic effect in CLRP-1 (Figure 4E). All these results indicated that CLRP-1 and CLSP-1 could increase the resistance to oxidative stress. Compared with the present polysaccharides, including *Angelica sinensis* polysaccharide and *Echinacea purpurea* polysaccharide (Zhuang et al., 2018; Hou et al., 2020), CLRP-1 and CLSP-1 had similar effects *in vitro*, which counteracts oxidative stress through decreasing MDA levels and increasing antioxidant enzyme activities.

The free radical scavenging ability of a polysaccharide and its function are mainly affected by the monosaccharide composition, molecular weight and glycoside bond configuration (Liu et al., 2020). The antioxidant activity and structural features of CLRP-1, CLSP-1 were compared, CLRP-1 had a better recovery effect and could improve activities of SOD and CAT more than CLSP-1. According to the above structure information, the *Mw*, AG-I and AG-II are distinctive, they could be connected with the activity of polysaccharides. The lower *Mw* the better activities (Lo et al., 2011), which may be a reason why CLRP-1 had better antioxidant demonstrates than CLSP-1. Furthermore, the length of HG backbone and AG chains of polysaccharides could also lead to the difference in activity. In our previous studies, we found that if the shorter HG backbone and AG chains, the polysaccharide would demonstrate the better activities (Huang et al., 2017; Yao et al., 2018). Compared with CLSP-1, CLRP-1 had a shorter HG backbone, which may be the reason that CLRP-1 had better activity than CLSP-1.

### 3.6 CLSP-1 protects *C. elegans* against thermal stress

**FIGURE 5:**
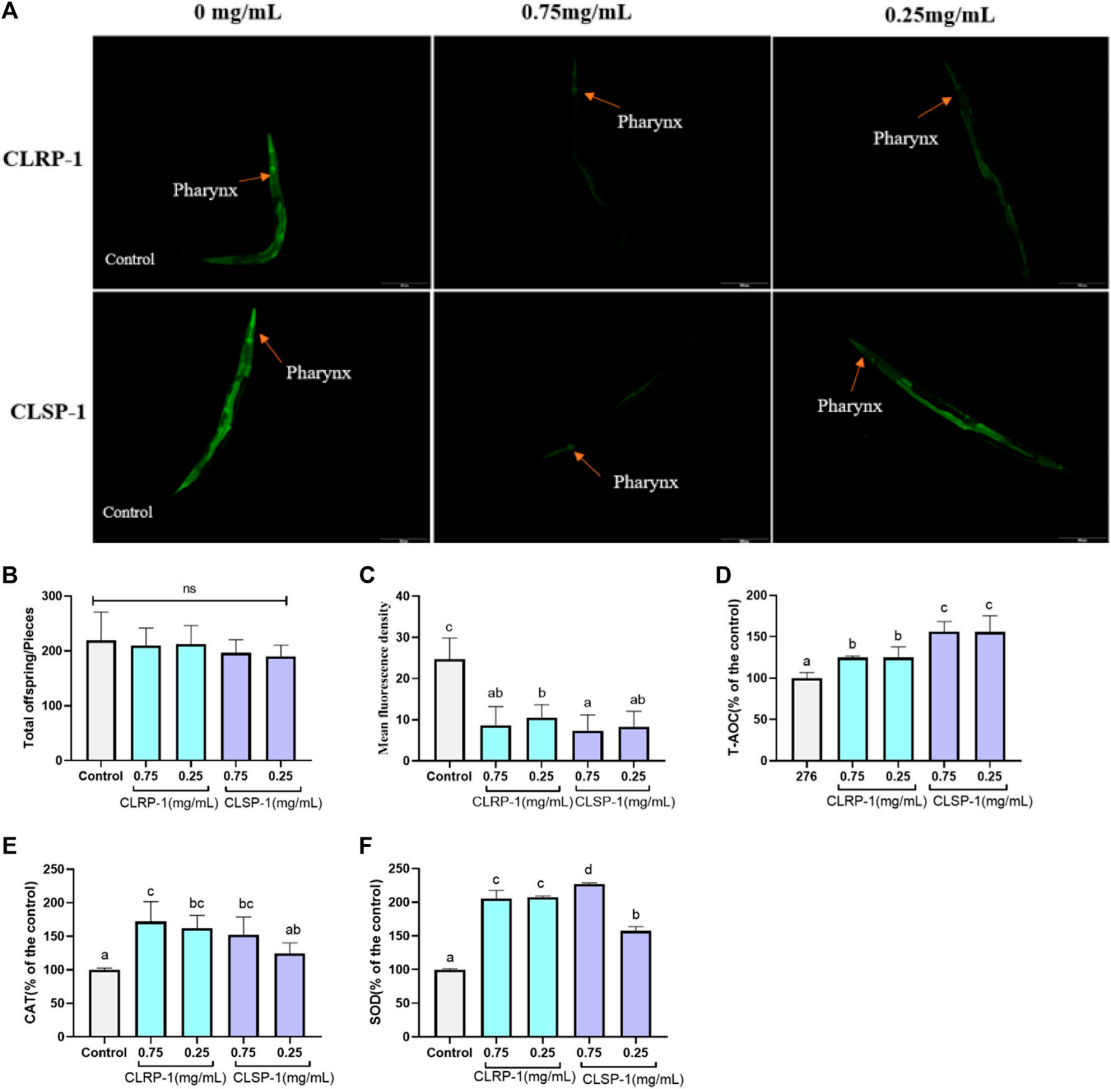
Effect of CLRP-1 and CLSP-1 on ROS level and antioxidant enzymes activities under thermal stress. **(A)** Fluorescence intensity of ROS. **(B)** CLRP-1 and CLSP-1 have no reproductive toxicity to normal *C. elegans.* **(C)** CLRP-1 and CLSP-1 could reduce ROS levels. **(D)** CLRP-1 and CLSP-1 could enhance T-AOC. **(E)** CLRP-1 and CLSP-1 could enhance CAT activities. **(F)** CLRP-1 and CLSP-1 could enhance SOD activities. ns indicates a statistically difference with *p* > 0.05. Bars with different lowercase letters **(a–d)** are significantly different (*p* < 0.05).

As an important model organism, *C. elegans* is widely used in drug screening and anti-oxidation and anti-aging (Zečić and Braeckman, 2020). As shown in Figure 5B, 0.75 mg/ml and 0.25 mg/ml of CLSP-1 and CLRP-1 all could not affect the total number of *C. elegans* progeny in comparison to the control group, indicating that CLRP-1and CLSP-1 under selected concentration did not affect the reproductive performance of *C. elegans*.

ROS, produced by various biochemical and physiological oxidative processes in the body, is also associated with numerous pathophysiological processes (Prasad et al., 2017). The heat stressor (35°C) could rapidly induce *C. elegans* to generate free radicals and subsequently increase intracellular oxidative stress. As shown in Figure 5, both concentrations of CLRP-1 and CLSP-1 significantly decreased ROS contents (*p <* 0.01), compared to the control group. These results indicated that the two polysaccharide fractions had the potential ability to scavenge free radicals *in vivo*.

To further verify whether CLRP-1 and CLSP-1 could increase the antioxidant enzyme levels in *C. elegans*, the antioxidant enzyme activities in *C. elegans* were determined. The results showed that both CLRP-1 and CLSP-1 displayed a better antioxidant activity in a concentration-dependent manner (Figures 5C–F). 0.75 mg/ml and 0.25 mg/ml of CLRP-1 could increase the level of T-AOC and CAT about by 25% and 70% (*p <* 0.05). Moreover, CLRP-1 could significantly accelerate the activity of SOD (*p* < 0.01). Similar results had been found in CLSP-1, 0.75 mg/ml of CLSP-1 pretreatment could remarkably increase SOD and CAT activities by 127% and 52%, respectively. T-AOC had a significant improvement as well (Figure 5D). All data above demonstrated that CLRP-1 and CLSP-1 could increase the antioxidant enzyme activities to decrease ROS levels in *C. elegans*, which were consistent with results of oxidative stress resistance *in vitro* (Figure 4).

The transparency of *C. elegans* renders them particularly favorable for the use of molecular probes, allowing for site-specific visualization of ROS formation, which is a significant advantage compared to fruit flies and mice (Labuschagne and Brenkman, 2013). Acid hydrolysates from *Auricularia auricular* polysaccharides could significantly increase the level of antioxidants and the expression of antioxidant-related genes to reduce ROS and MDA contents in *C. elegans* (Fang et al., 2019). Zhang et al. (2016) also showed that *Dictyophora indusiata* polysaccharide could increase SOD activity to decrease ROS and MDA contents in *C. elegans*. Similar results were found in this study, that both different concentrations of CLRP-1 and CLSP-1 could significantly reduce the ROS level in *C. elegans* after thermal stress. T-AOC level had been significantly improved as well as the activities of SOD and CAT, which was consistent with the effects of two polysaccharides on the IPEC-J2 *in vitro*. Taken together, CLRP-1 and CLSP-1 showed potential antioxidant activities *in vivo* and *in vitro*, which was mainly due to the high content of galacturonic acid (Chen et al., 2019). Meanwhile, pectin contains hydroxyl groups that may display antioxidant capacity. In addition, in monosaccharide, such as Rha, also played an important role in antioxidative (Jiang et al., 2013). The lower molecular weight of CLRP-1 had better antioxidant activity *in vitro* and *in vivo*, which was similar to previous reports (Xing et al., 2008; Zhou et al., 2012).

### 3.7 Quantification of HSP-16.2::green fluorescent protein expression

**FIGURE 6:**
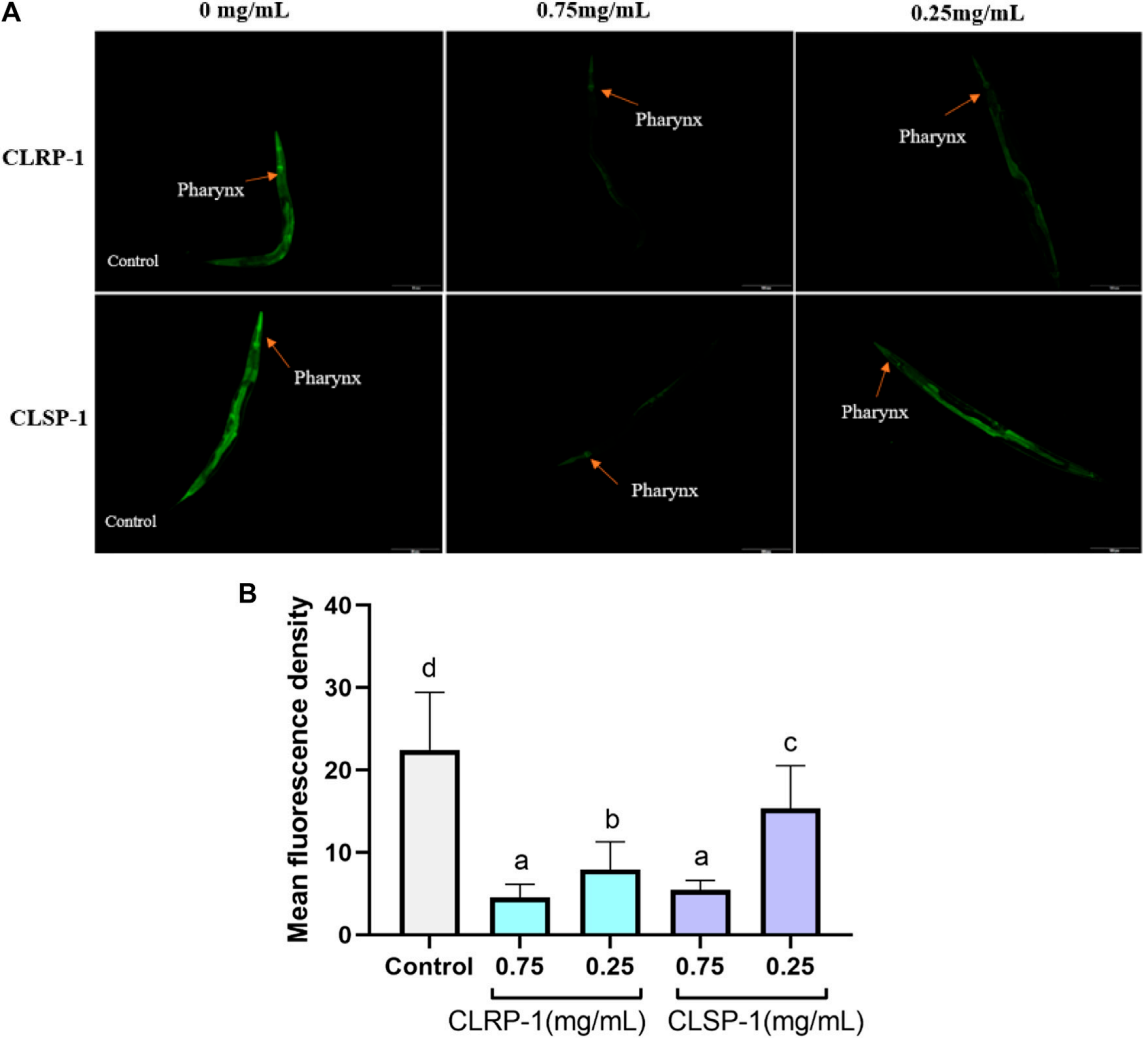
Effect of CLRP-1 and CLSP-1 on the expression of HSP-16.2:GFP under thermal stress. **(A)** The HSP-16.2::GFP expression. **(B)** CLRP-1 and CLSP-1 could reduce the on the HSP-16.2::GFP expression under thermal stress. Bars with different lowercase letters **(a–c)** are significantly different (*p* < 0.05).

TJ375 mutants have *hsp-16.2* promoter coupled to green fluorescent protein (GFP) reporter which can be induced by thermal stress and oxidative stress (Strayer et al., 2003). Under exposure to thermal stress, the strong fluorescent signal of HSP-16.2::GFP could be observed by a fluorescence microscope. As shown in Figure 6, both 0.75 mg/ml and 0.25 mg/ml of CLRP-1 and CLSP-1 could reduce significantly the fluorescence intensity of *C. elegans* compared to the control group after thermal stress, and 0.75 mg/ml of CLRP-1 presented the best effect, suggesting that CLRP-1 and CLSP-1 could regulate the HSP-16.2::GFP expression to increase thermal stress resistance.

Epigallocatechin gallate down-regulated *hsp-16.1* and *hsp-16.2* under oxidative stress, protecting the macromolecules from oxidation damage (Abbas and Wink, 2010). Betalains decreased the expression of HSP-16.2::GFP to resist oxidative stress (Guerrero-Rubio et al., 2019). In the present study, the heat stressor-induced expression of HSP-16.2::GFP was suppressed by CLRP-1 and CLSP-1, which proved that two polysaccharides had a protective effect against thermal stress and CLRP-1 had a better effect than CLSP-1.

### 3.8 DAF-16 transcription factor intracellular localization

**FIGURE 7:**
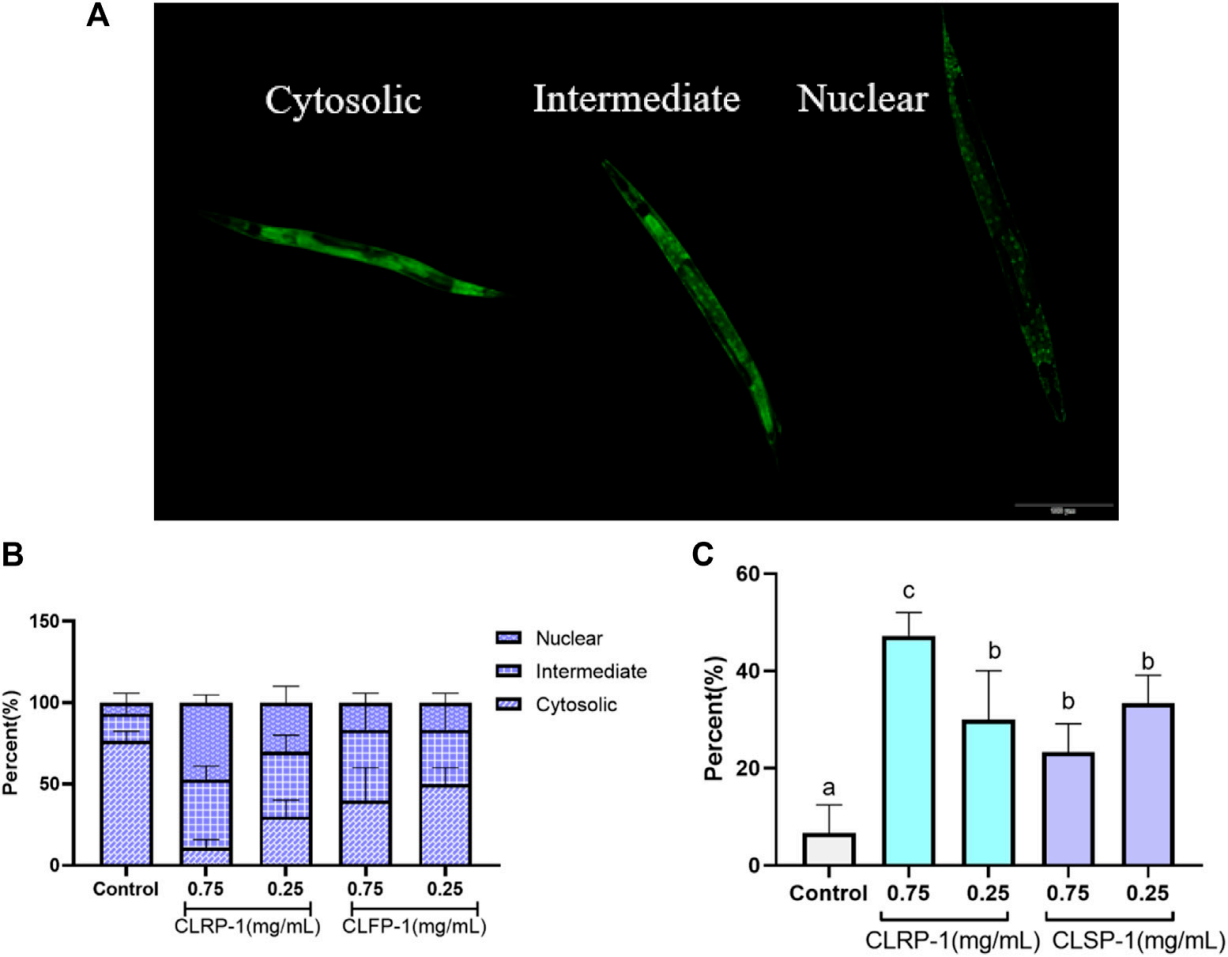
Effect of CLRP-1 and CLSP-1 on the transcription factor of DAF-16 under thermal stress. **(A)** The localization of transcription factor of DAF-16. **(B)** The localization of DAF-16. **(C)** The percentage of nuclear localization. Bars with different lowercase letters **(a–c)** are significantly different (*p* < 0.05).

*C. elegans* strain TJ356 (DAF-16::GFP) expresses green fluorescent protein fused to the transcription factor DAF-16. The nuclear localization of DAF-16 was a necessary prerequisite for the transcriptional activation of a broad spectrum of target genes, including antioxidant enzymes such as SOD-3 (Murphy et al., 2003; Oh et al., 2006; Lin et al., 2019). As shown in Figure 7A, the following figures respectively showed the localization of transcription factor of DAF-16 in *C. elegans*. The results found that CLRP-1 and CLSP-1 could significantly promote DAF-16 to enter the nucleus in the body of *C. elegans*, and 0.75 mg/ml CLRP-1 had the best effect (Figures 7B,C).

Previous studies had shown that DAF-16 is closely related to aging and oxidative stress (Oh et al., 2006; Zečić and Braeckman, 2020). Under stress, the transcription factor of DAF-16 translocated into the nucleus and target genes of antioxidant enzymes will increase their expression, which improved the antioxidant performance of *C. elegans*. For example, Liangyi Gao, a traditional Chinese medicine, had robust and reproducible life-prolonging in *C. elegans* via DAF-16/FOXO regulation (Zeng et al., 2019). Treatment with blueberry extract resulted in up-regulation of genes related to antioxidant systems, including SOD-3, CTL-1, MEV-1, SKN-1, and DAF-16 (Wang et al., 2018). In this study, CLRP-1 and CLSP-1 could promote the transcription factor DAF-16 into the nucleus, thereby enhancing the antioxidant properties of *C. elegans*.

## 4 Conclusion

Three purified pectic polysaccharide fractions were obtained by DEAE ion exchange chromatography and gel filtration from the different plant parts of *C. pilosula* var. *modesta* (Nannf.) L. T. Shen. The results of cellular antioxidant activity in three fractions showed that CLFP-1 had the lowest activity. CLRP-1 and CLSP-1 could protect cells against oxidative stress and could increase the level of antioxidant enzymes *in vitro* and *in vivo*. The results of structural elucidation showed that the *Mw* of CLSP-1 is higher than CLRP-1, and CLRP-1 has richer monosaccharide composition. In the linear region of the two polysaccharides are mainly represented by the segments of 1,4-α-D-GalA (partially methyl-esterified and some of the residues are acetylated). The branched region is represented by RG-I with AG-II side chain on position four of Rha, and AG-I side chain on position two of GalA. Therefore, two polysaccharides were typical pectin polysaccharides and the main features of fraction CLRP-1 and CLSP-1 were with a long HG region, with arabinogalactan type I (AG-I) and arabinogalactan type II (AG-II) as the side chains. The expression of HSP-16.2::GFP was suppressed significantly in *C. elegans* and nuclear localization of DAF-16 transcription factor were increased by giving CLRP-1 and CLSP-1. It provided some evidence for the development and utilization of the *C. pilosula* var*. modesta* (Nannf.) L. T. Shen resources.

## Data Availability

The original contributions presented in the study are included in the article/Supplementary Material, further inquiries can be directed to the corresponding authors.
