# Supplementary material for: Structural features and antioxidant activities of polysaccharides from different parts of Codonopsis pilosula var. modesta (Nannf.) L. T. Shen
Source: Front Pharmacol. 2022 Aug 24;13:937581. doi: 10.3389/fphar.2022.937581 (PMC9449496; doi:10.3389/fphar.2022.937581)
Supplement: Supplementary file 1 [file DataSheet1.ZIP › Chromatogram of high performance size exclusion chromatography.pdf]

# Supplementary date

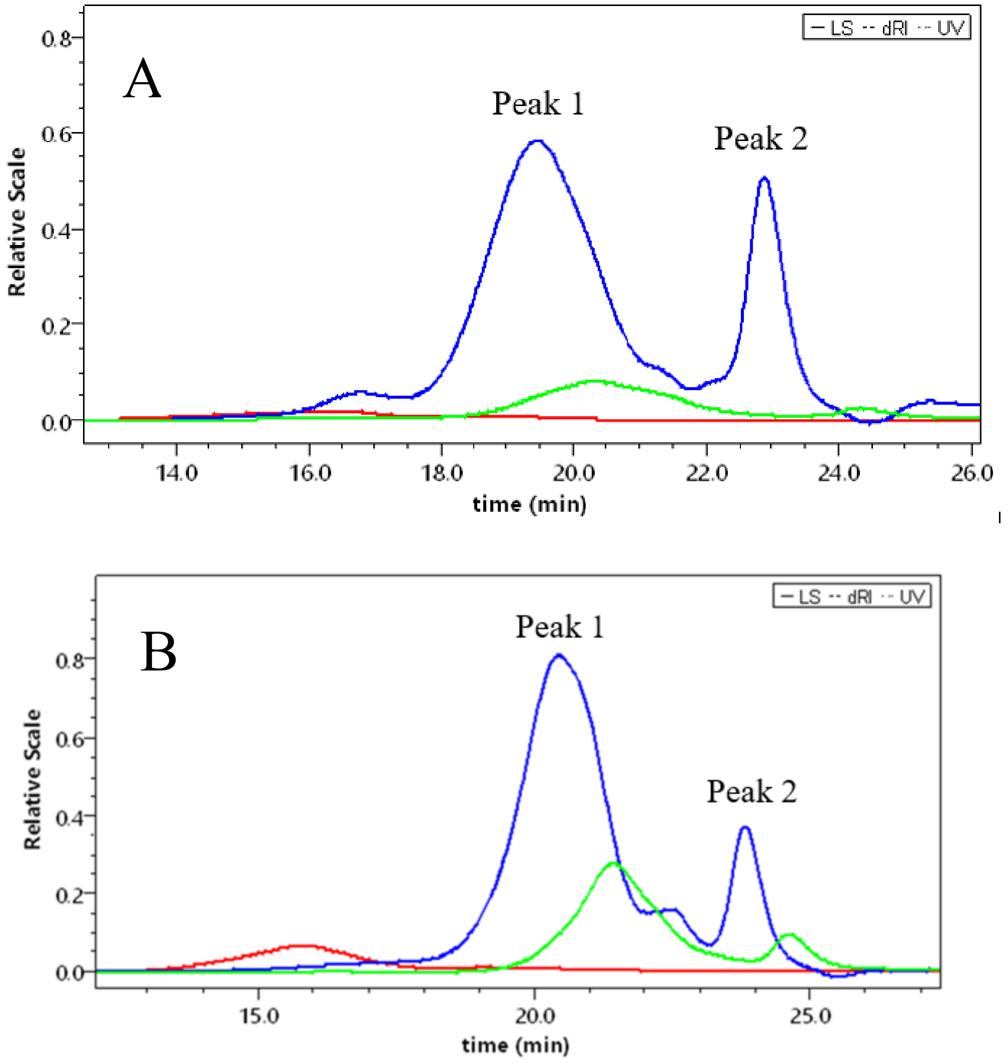


**Fig.S1.** Chromatogram of high performance size exclusion chromatography of (A) CLRP-1 and (B) CLSP-1. Peak 1 is the peak of sample; peak 2 is the peak of NaCl solvent. The blue line is the signal of RI, the green line is the signal of UV and the red line is the signal of LS.
