# Supplementary material for: Structural features and antioxidant activities of polysaccharides from different parts of Codonopsis pilosula var. modesta (Nannf.) L. T. Shen
Source: Front Pharmacol. 2022 Aug 24;13:937581. doi: 10.3389/fphar.2022.937581 (PMC9449496; doi:10.3389/fphar.2022.937581)

GC-MS data for Sample CLSP-1

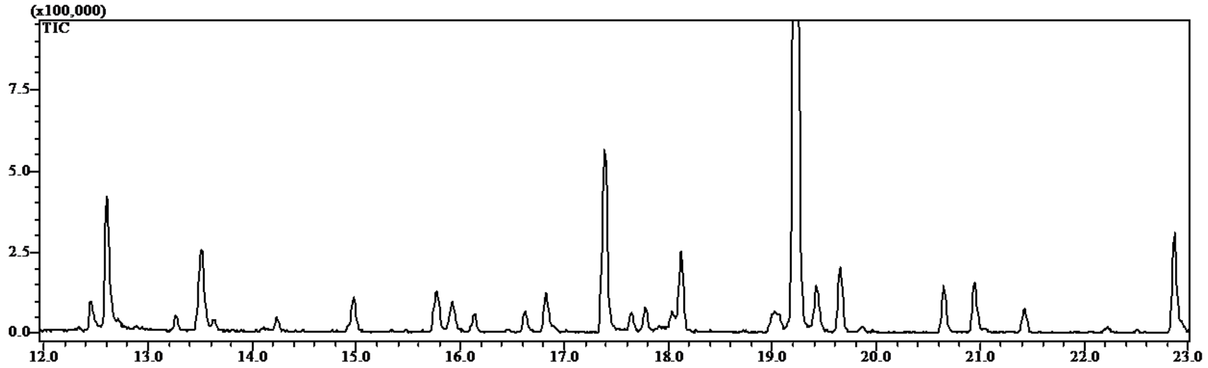

12.608 min, T-Ara*f*

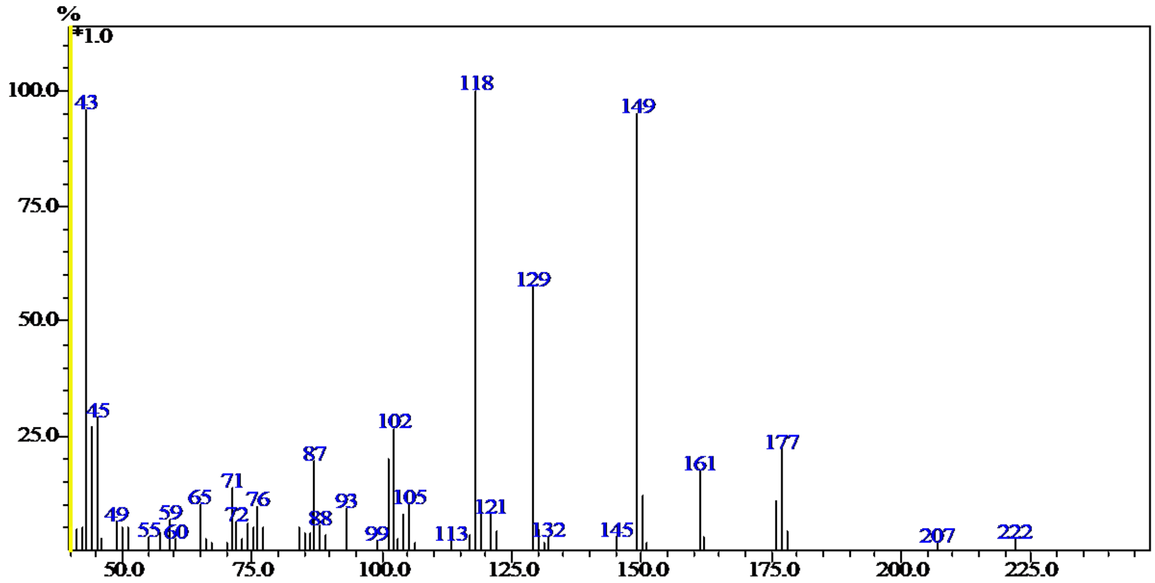

14.242 min, T-Rha*p*

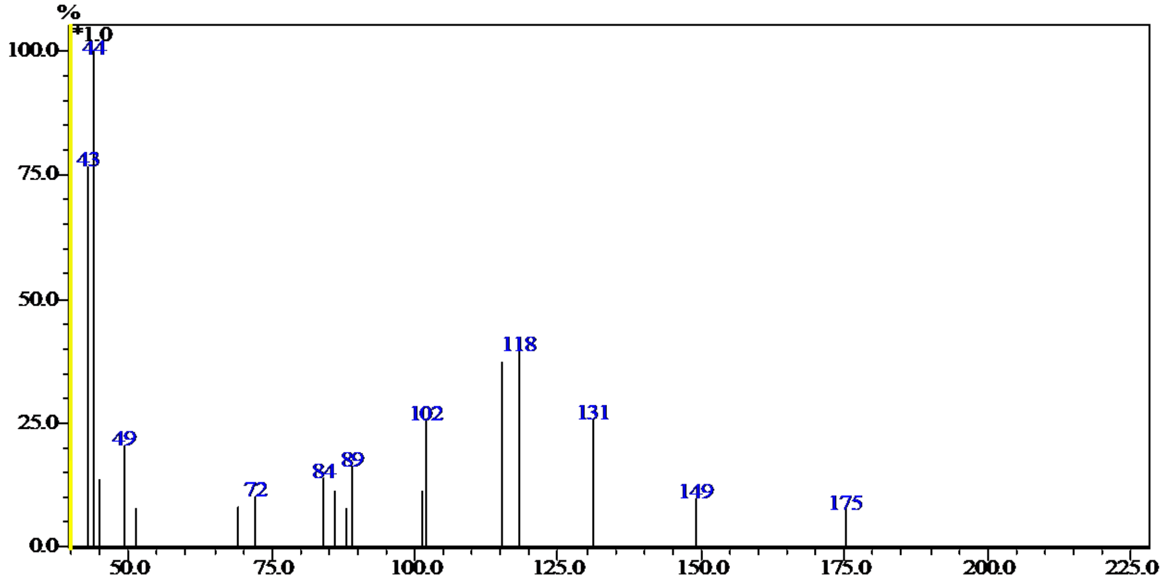

14.924 min, 1→2 Ara*f*

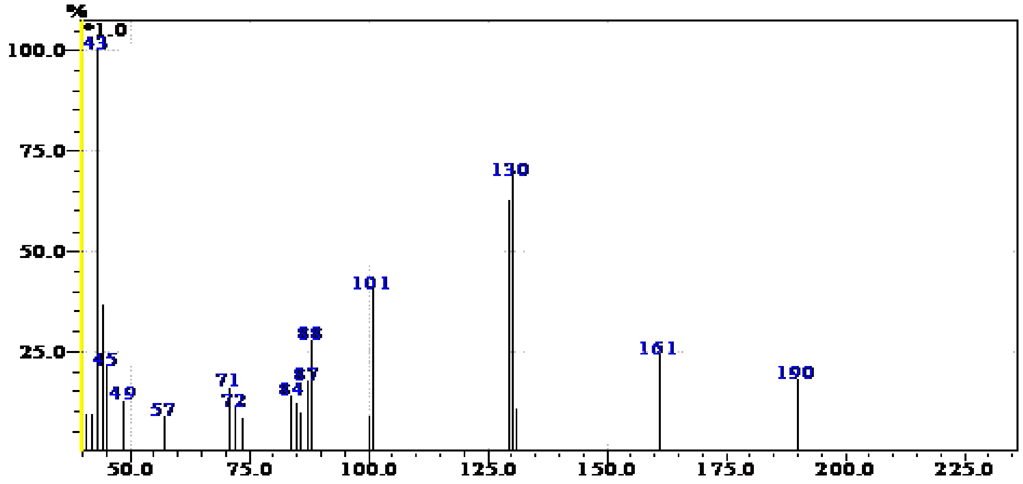

14.975 min 1→3 Ara*f*

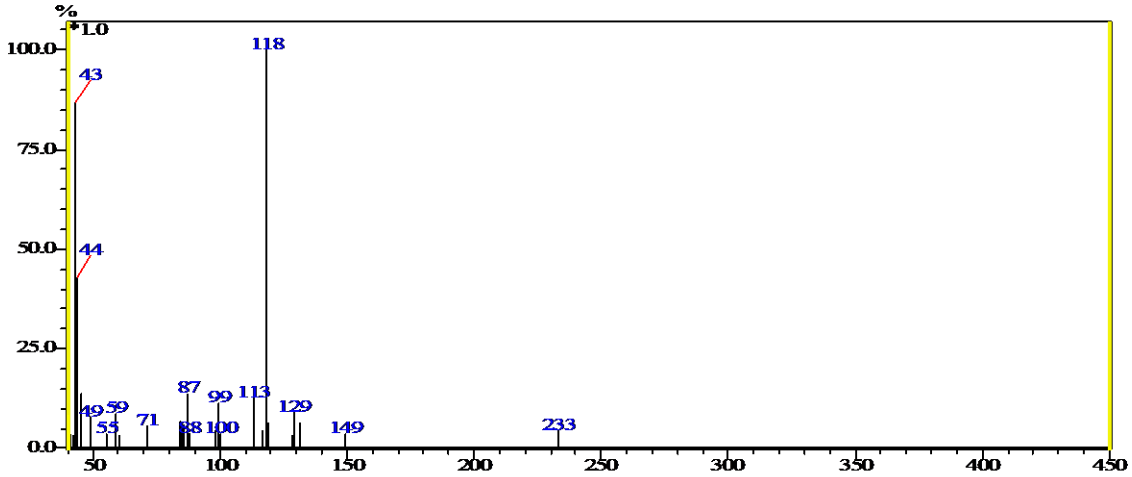

15.775 min, 1→2 Rha*p*

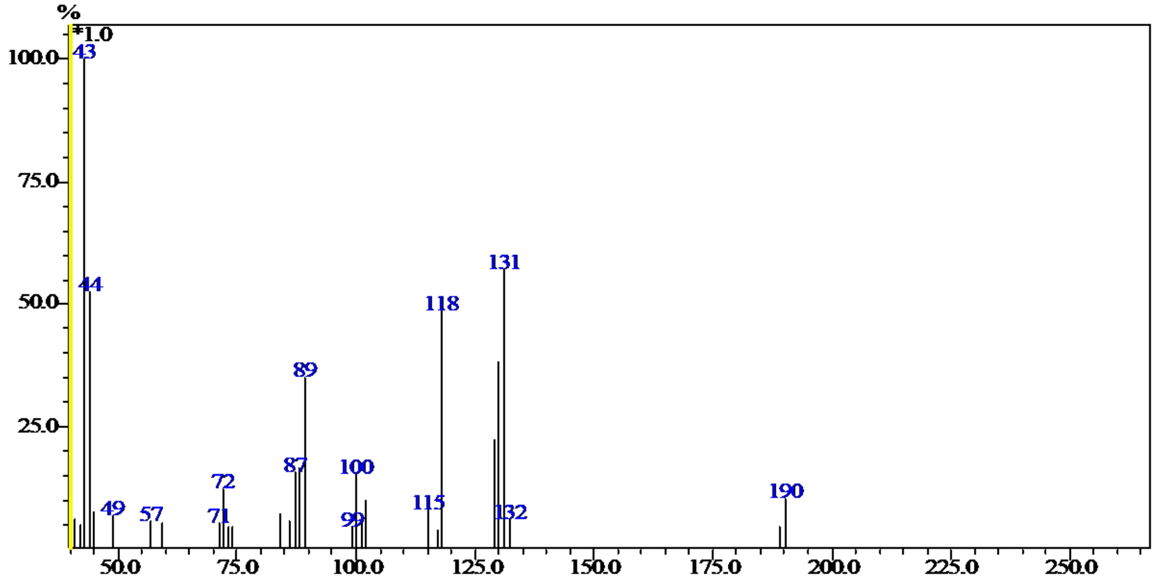

17.392 min, T-Gal*p* and T-GalA*p*

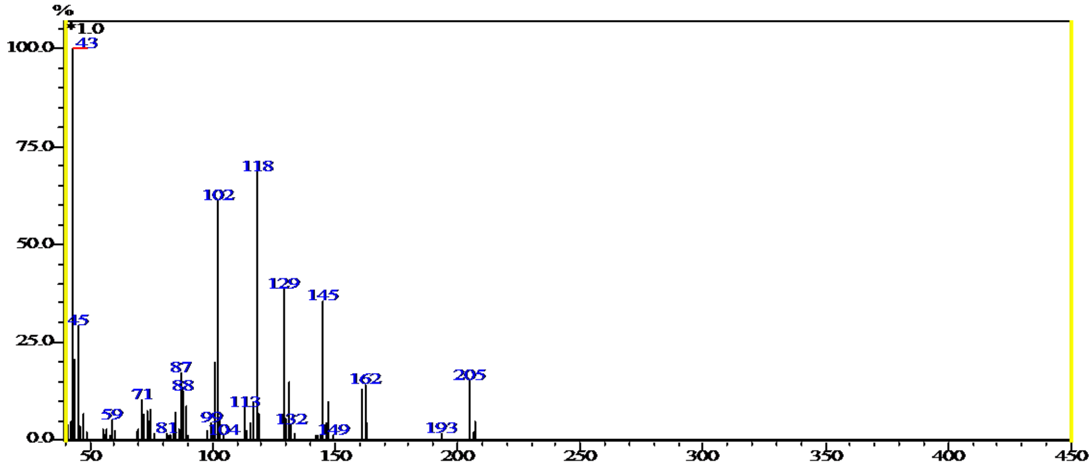

17.783 min, 1→3,5 Ara*f*

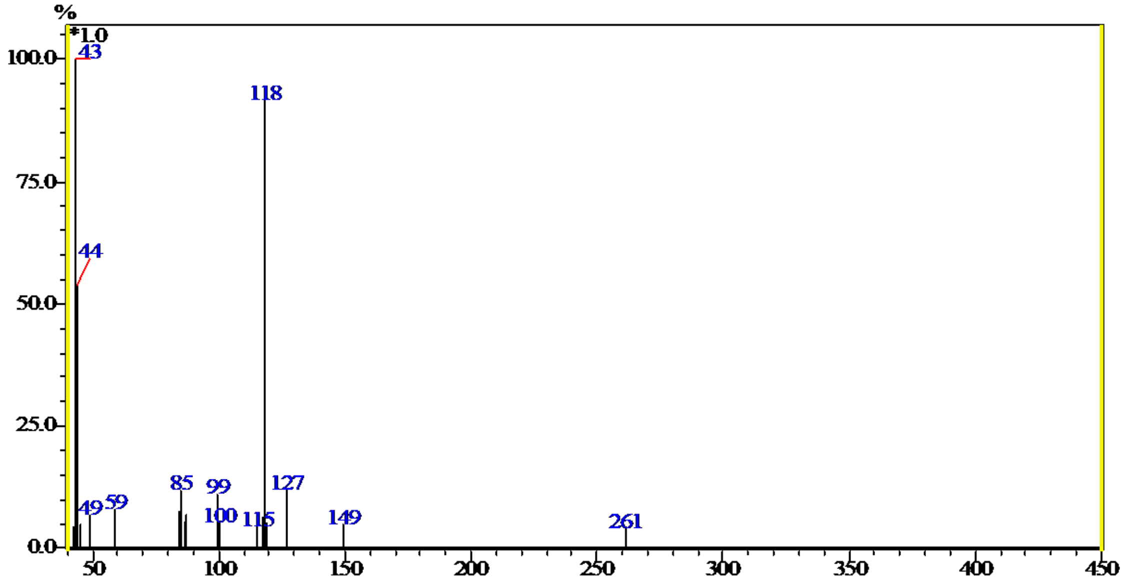

18.125 min, 1→2,4 Rha*p*

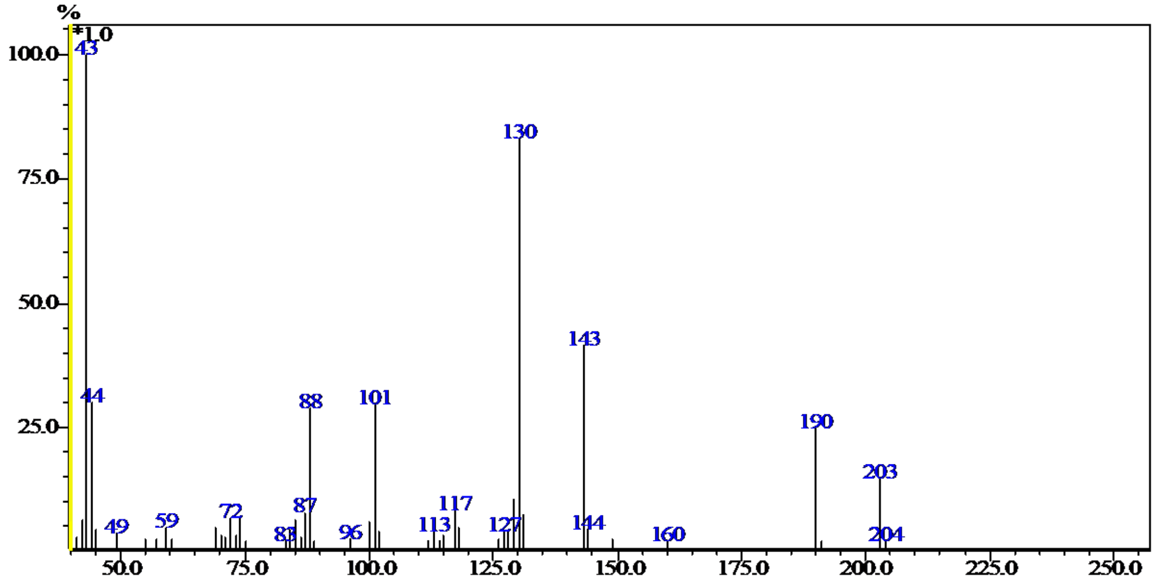

19.225 min 1→4 Gal*p* and 1→4GalA*p*

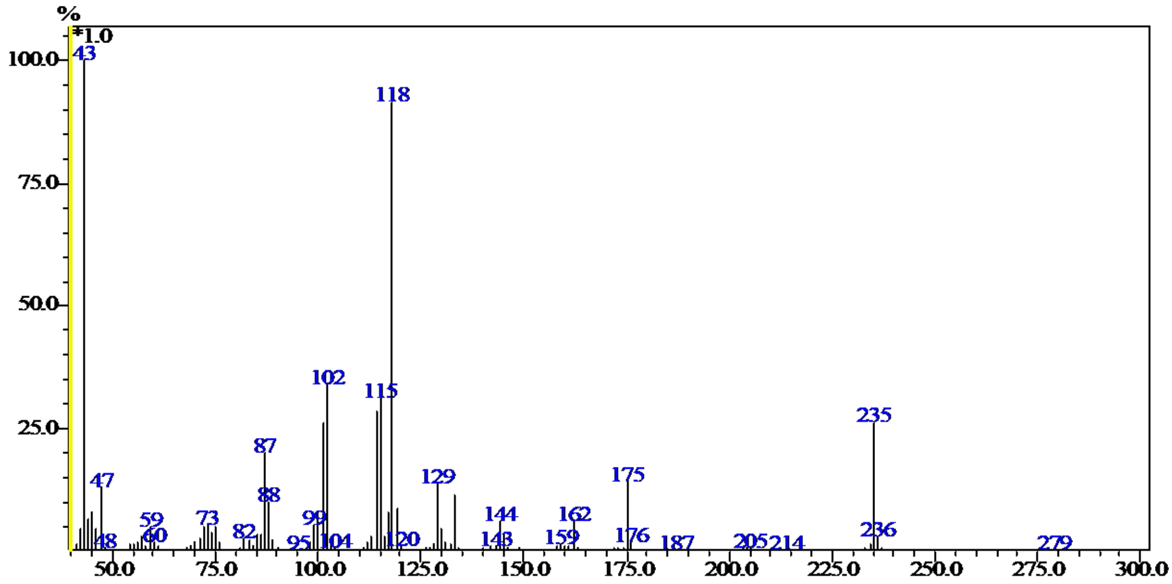

19.650 min, 1→3 Gal*p*

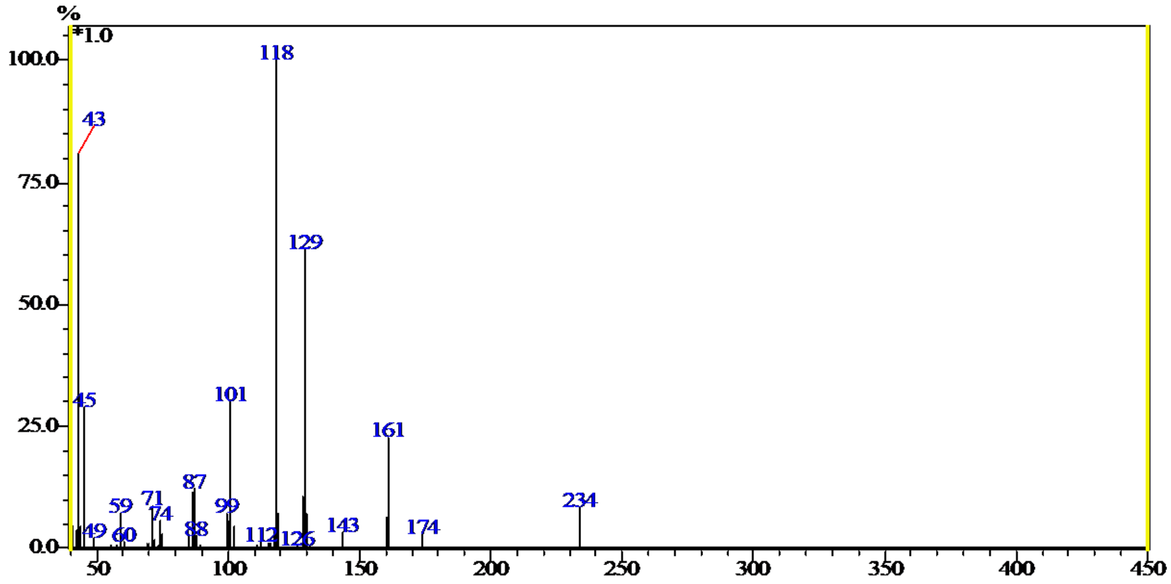

20.650 min 1→6 Gal*p*

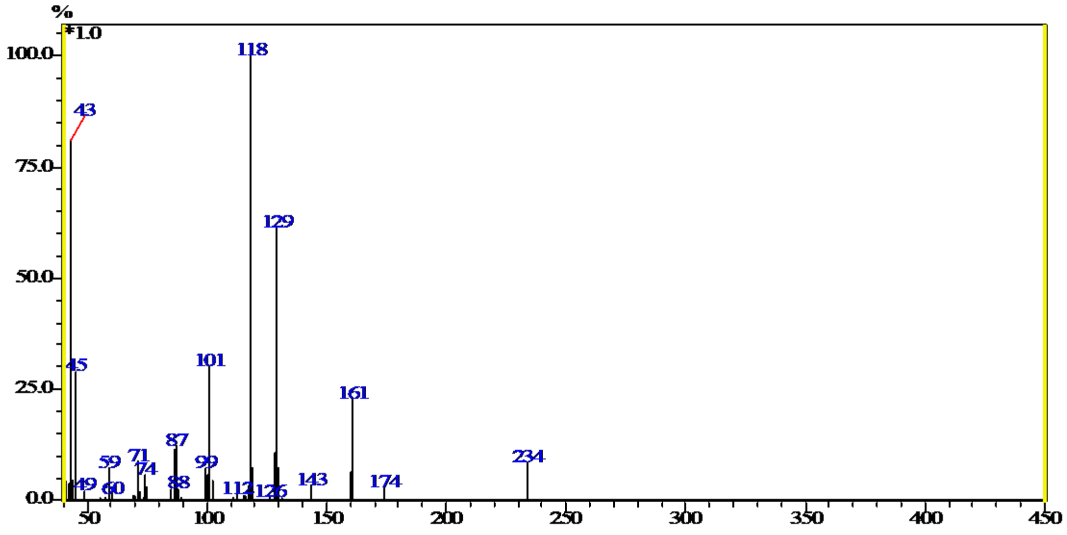

20.942 min 1→3,4 GalA

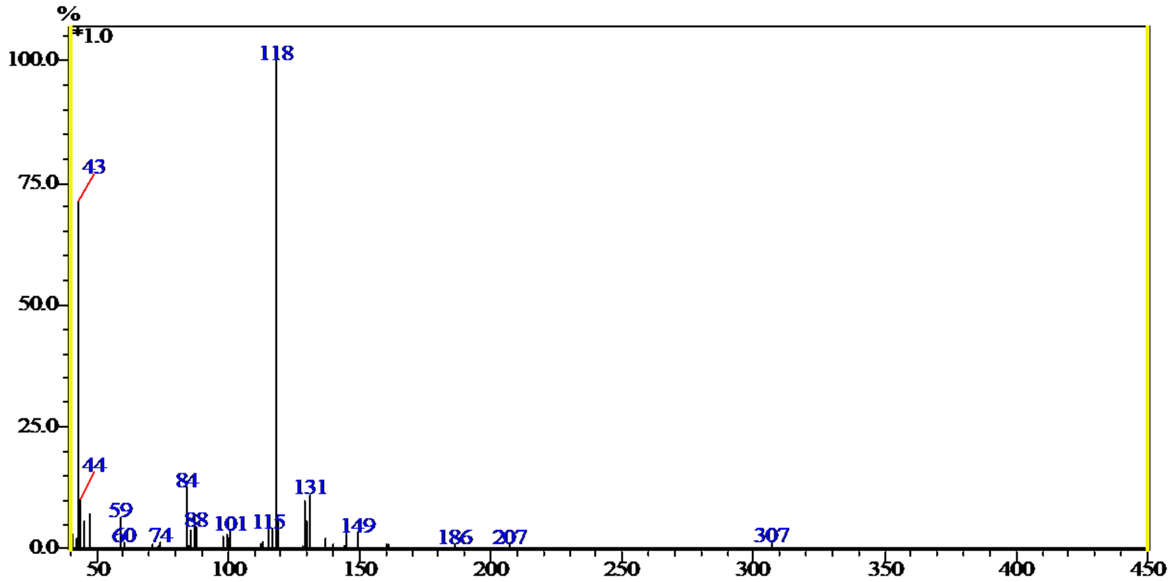

22.864 min, 1→3,6 Gal*p*

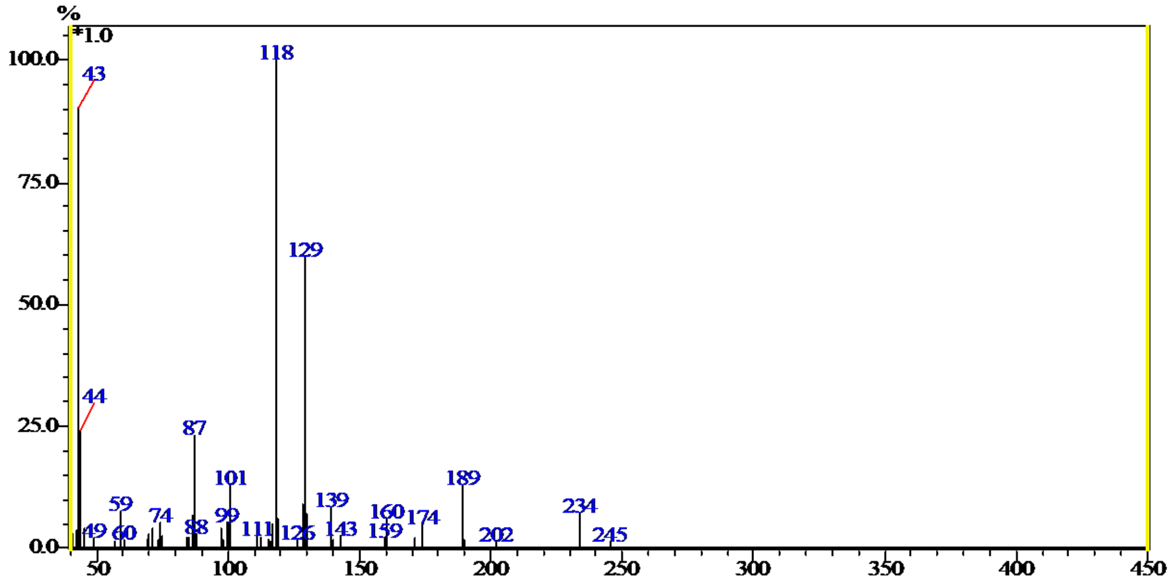

Supplement: Supplementary file 1 [file DataSheet1.ZIP › GC-MS data for Sample CLSP-1.pdf]
