# Supplementary material for: Structural features and antioxidant activities of polysaccharides from different parts of Codonopsis pilosula var. modesta (Nannf.) L. T. Shen
Source: Front Pharmacol. 2022 Aug 24;13:937581. doi: 10.3389/fphar.2022.937581 (PMC9449496; doi:10.3389/fphar.2022.937581)

GC-MS data for Sample CLRP-1

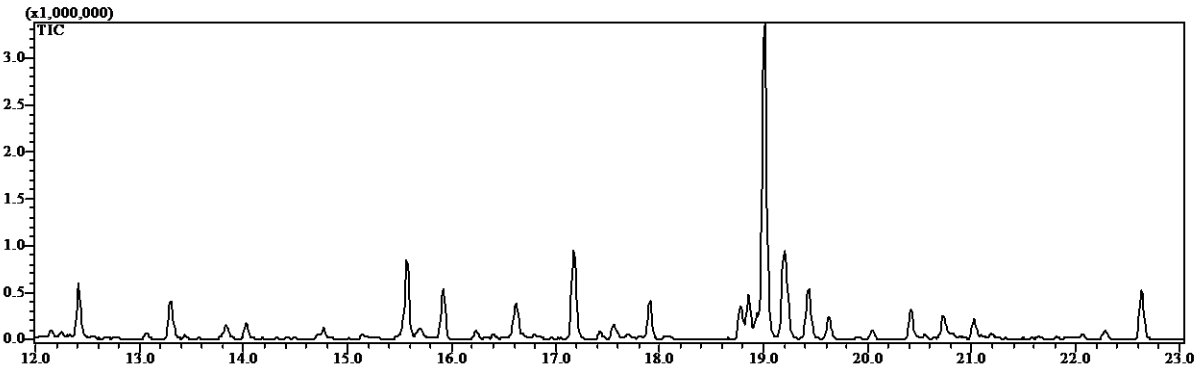

12.408 min, T-Ara*f*

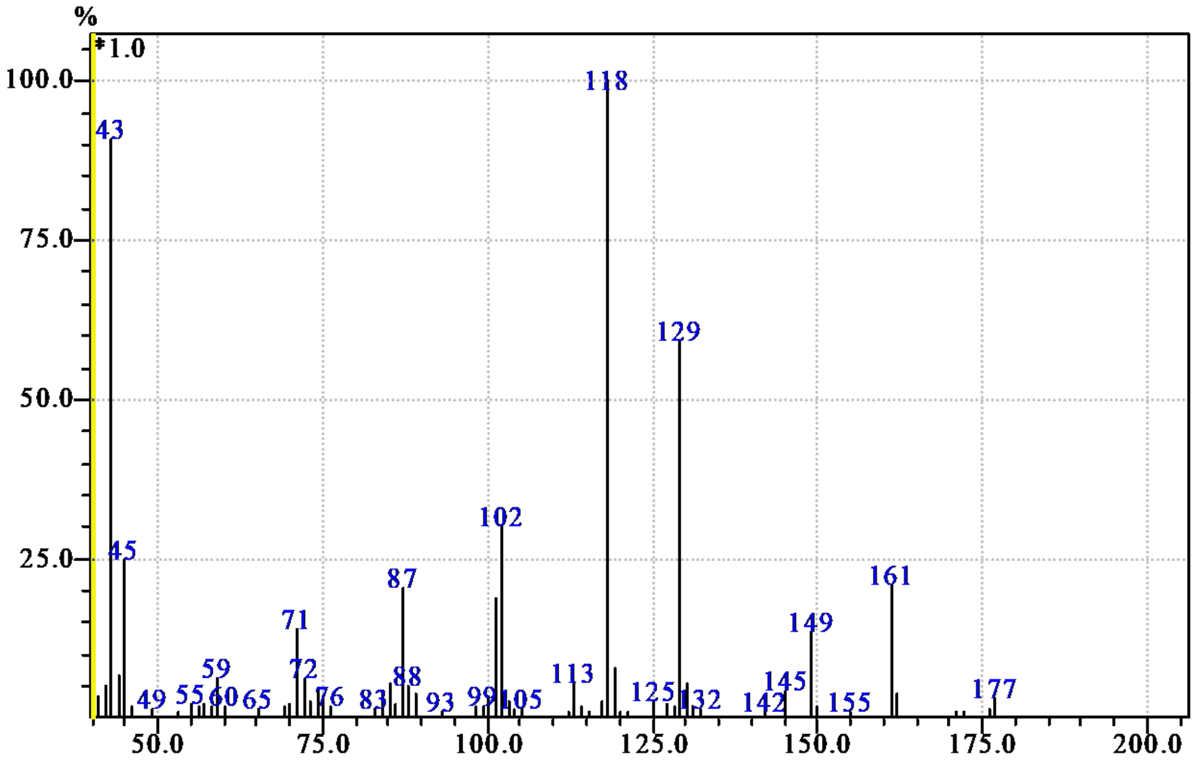

13.300 min, T-Xyl*p* and T-Rha*p*

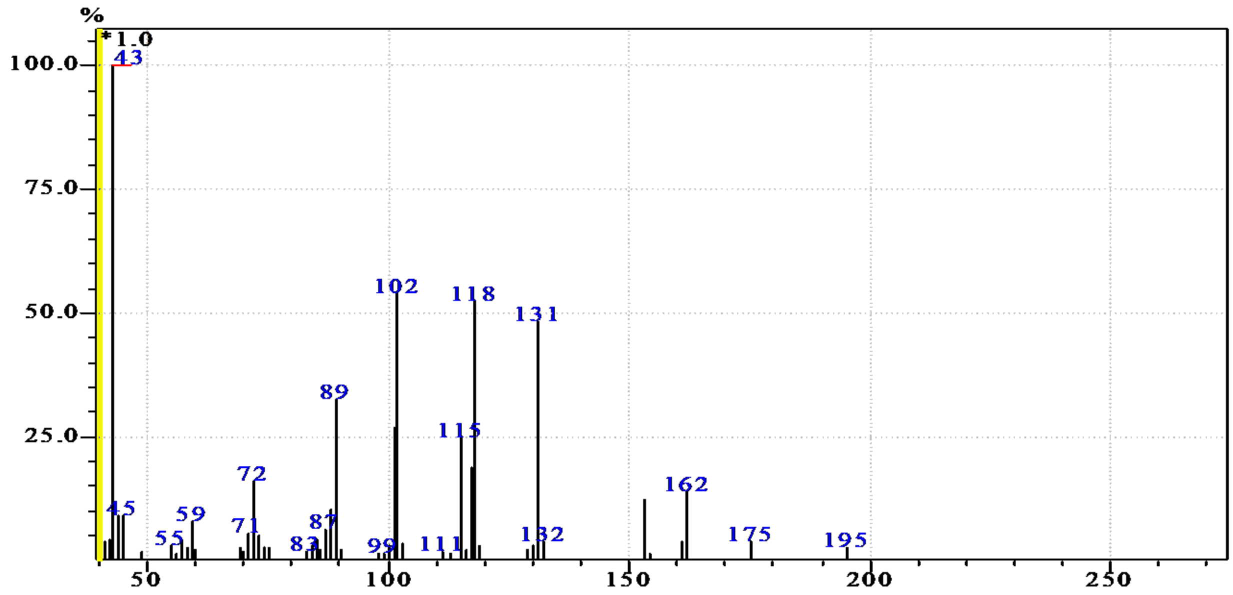

14.025 min, T-Fuc*p*

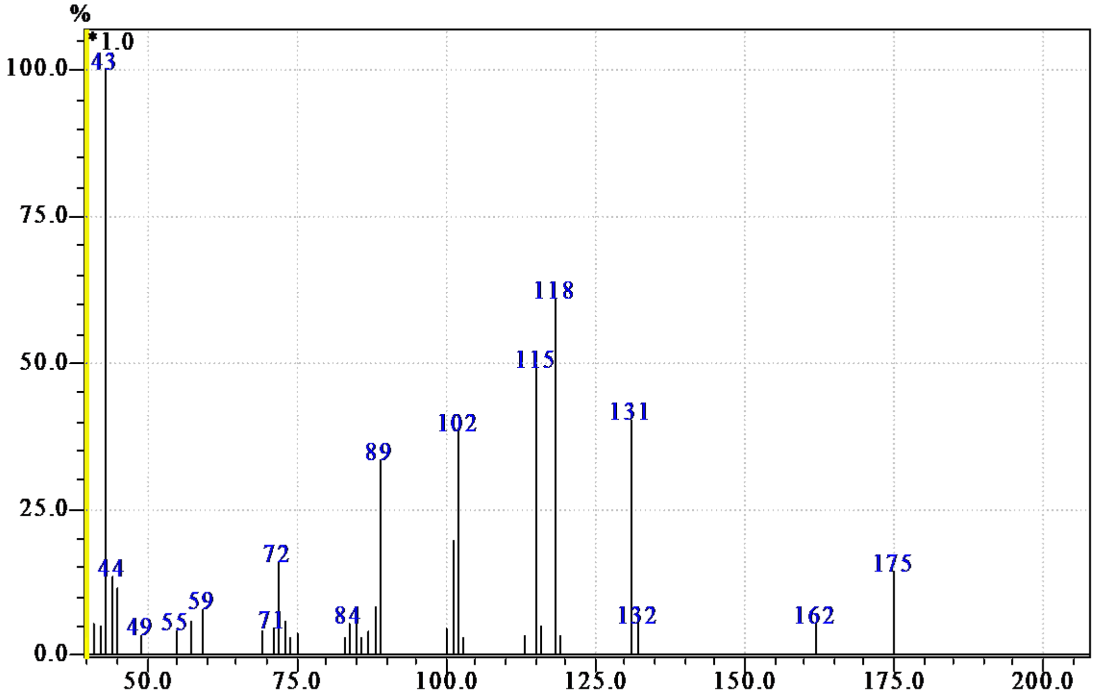

15.533 min 1→5 Ara*f*

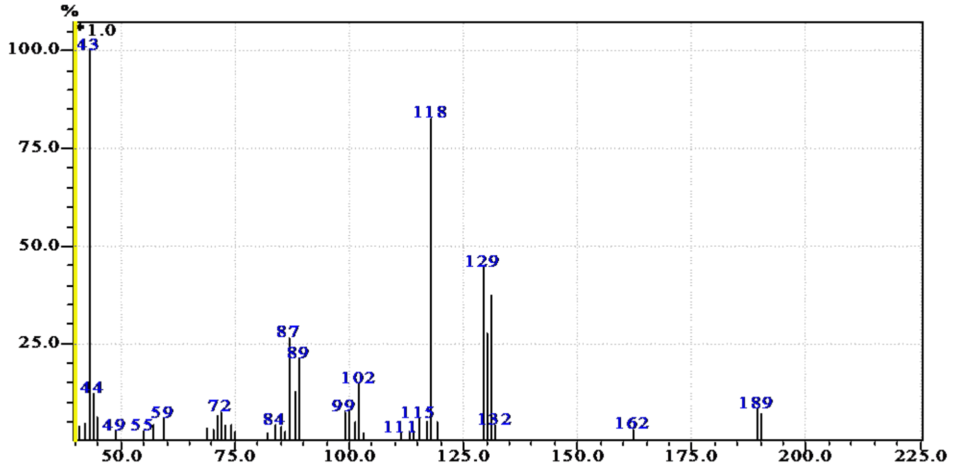

15.592 min, 1→2 Rha*p*

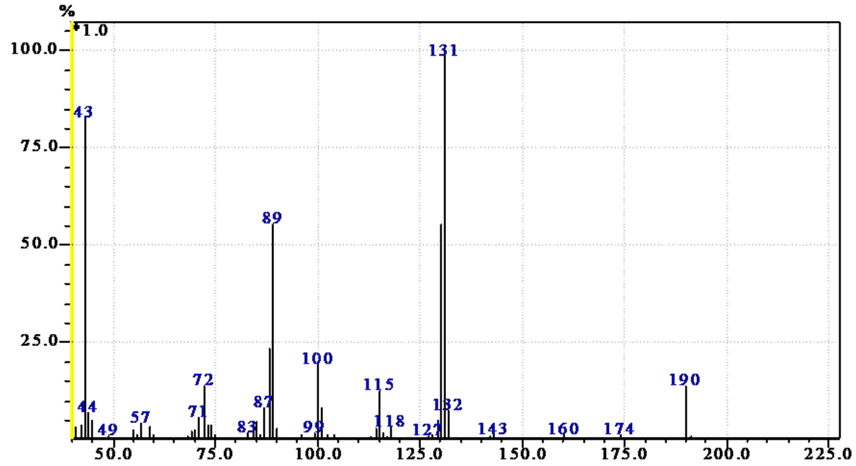

15.925 min, 1→3 Rha*p*

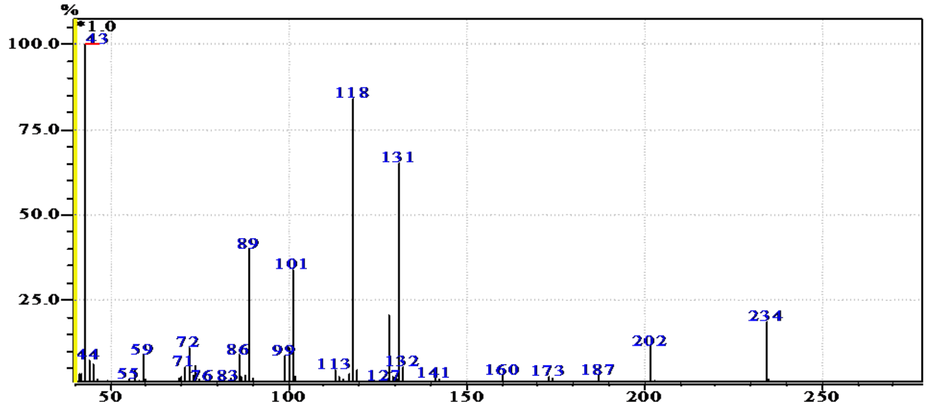

16.625 min, T-Glc*p* and T-GlcA*p*

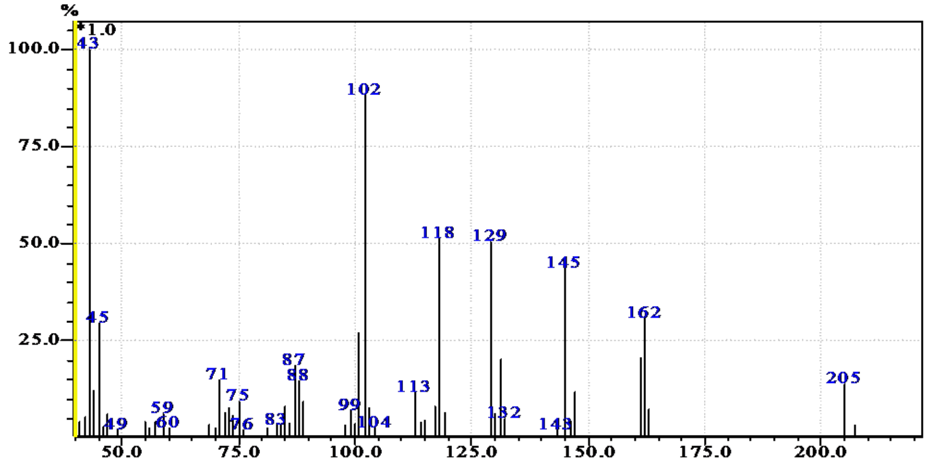

17.175 min, T-Gal*p* and T-GalA*p*

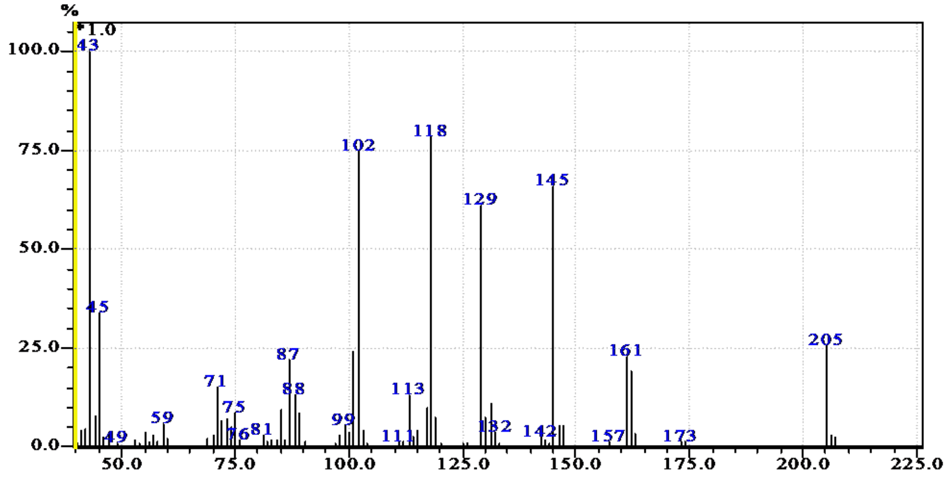

17.558 min, 1→3, 5 Ara*f*

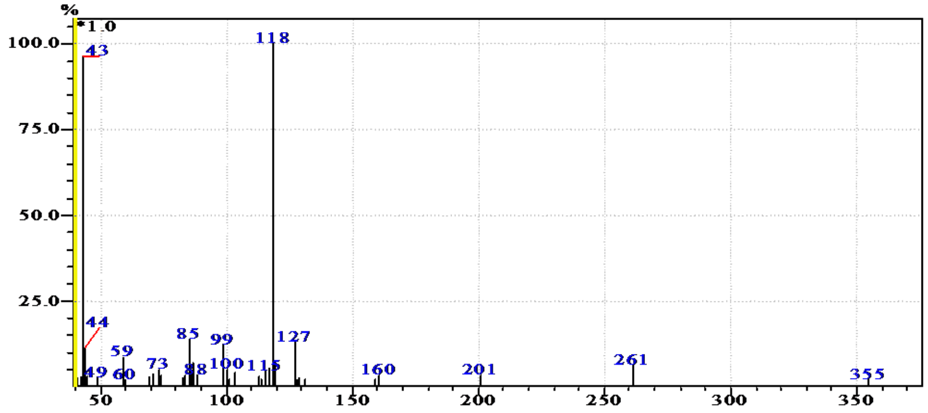

17.900 min, 1→2, 4 Rha*p*

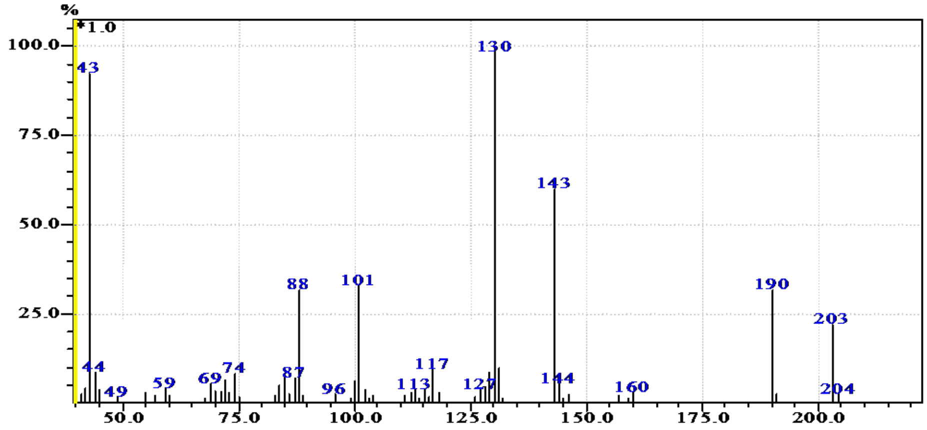

18.780 min, 1→2 Man*p*

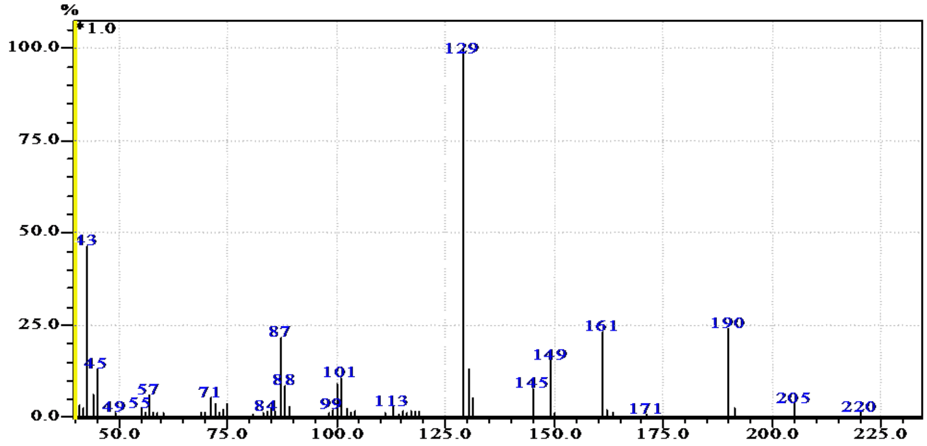

19.008 min, 1→4 Gal*p* and 1→4 GalA*p*

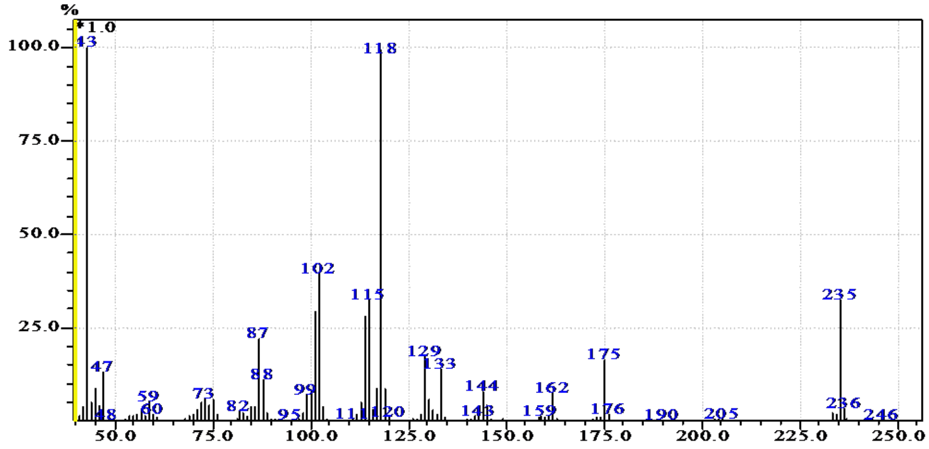

19.208 min, 1→4 Glc*p*

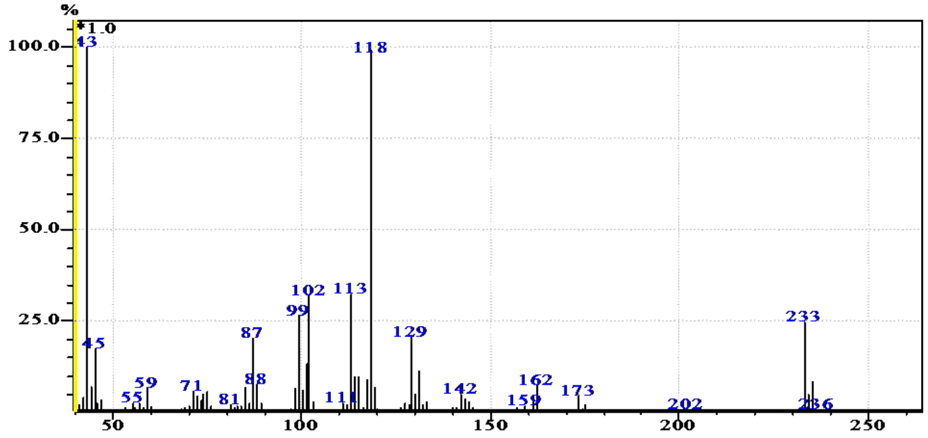

19.417min, 1→3 Gal*p*

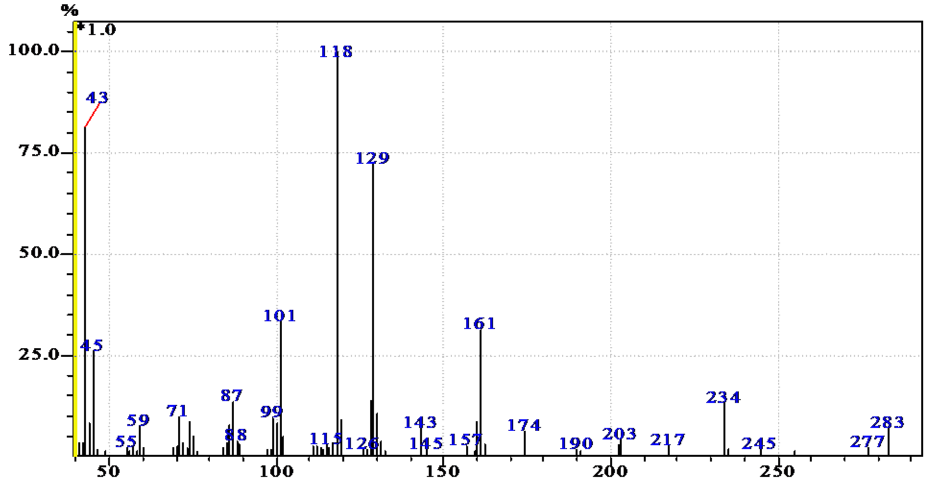

19.625 min, 1→6 Glc*p*

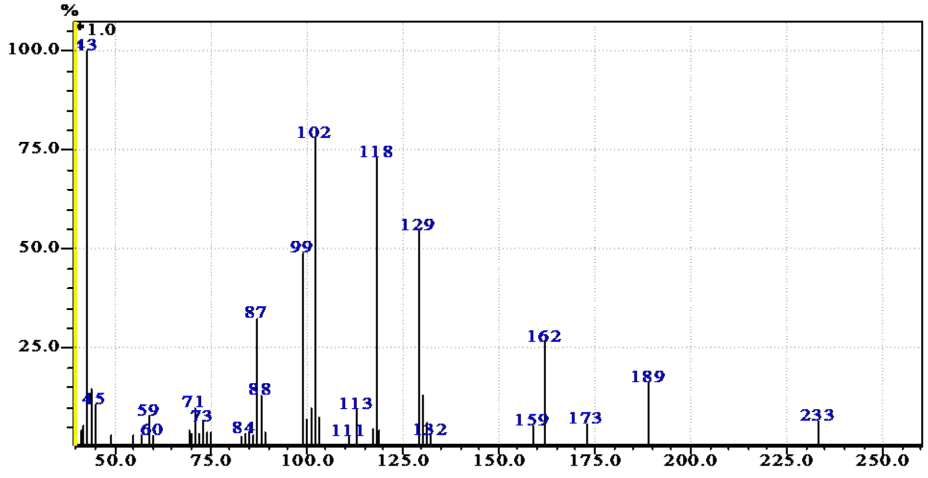

20.425 min, 1→6 Gal*p*

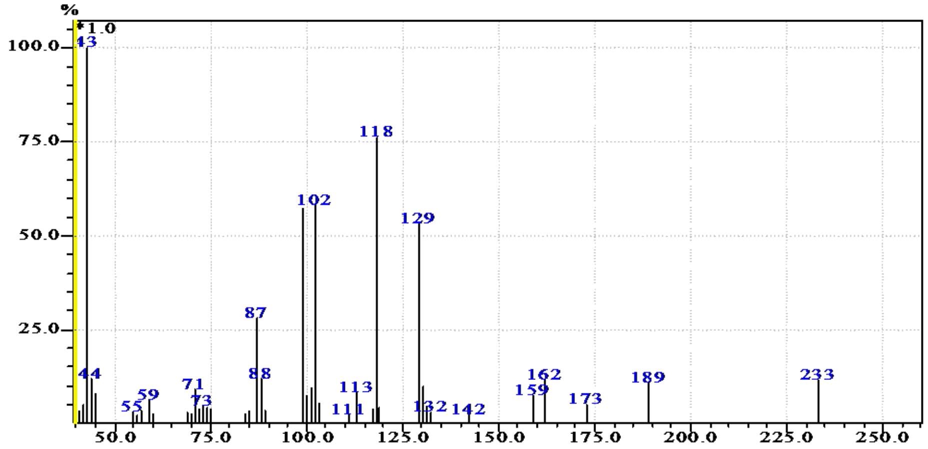

20.717 min, 1→3, 4 Gal*p* and 1→3, 4 GalA*p*

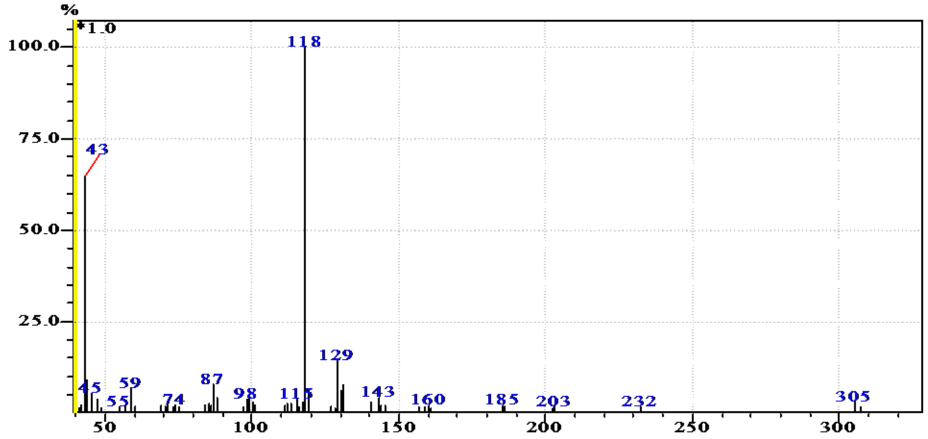

22.625 min, 1→3, 6 Gal*p*

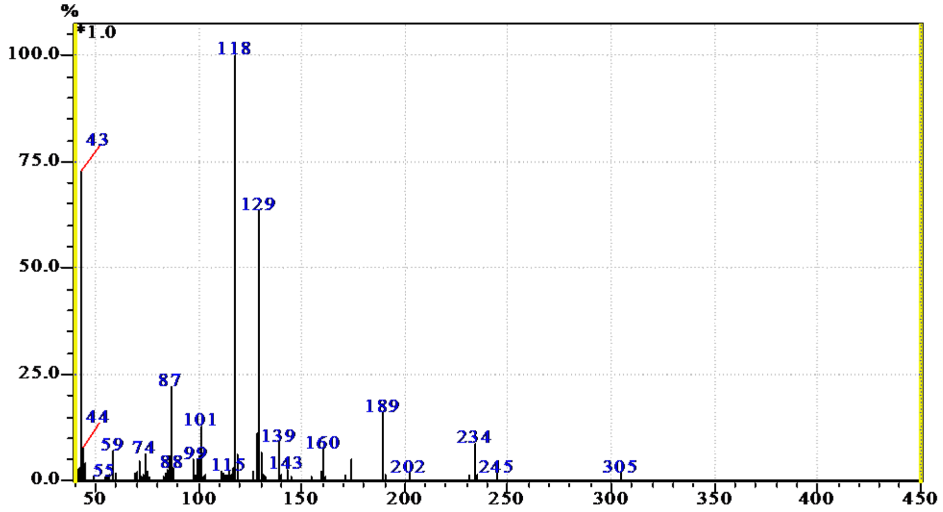

Supplement: Supplementary file 1 [file DataSheet1.ZIP › GC-MS data for Sample CLRP-1.pdf]
